# Effectiveness of phosphate binders on mortality and cardiovascular disease in end-stage renal disease patients with hyperphosphatemia: a multicenter real-world cohort study

**DOI:** 10.1186/s12882-025-04058-7

**Published:** 2025-03-10

**Authors:** Kamolpat Chaiyakittisopon, Oraluck Pattanaprateep, Wanchana Ponthongmak, Patratorn Kunakorntham, Anan Chuasuwan, Atiporn Ingsathit, Gareth J. Mckay, John Attia, Ammarin Thakkinstian

**Affiliations:** 1 Department of Clinical Epidemiology and Biostatistics, Faculty of Medicine, Ramathibodi Hospital, Mahidol University, 3rd Floor, Research Center Building, 270 RAMA VI Road. Ratchathewi, Bangkok, 10400 Thailand; 2 Department of Health Consumer Protection and Pharmacy Administration, Faculty of Pharmacy, Silpakorn University, Nakhon Pathom, Thailand; 3 Health Informatics Analyst Data Health for Analysis Informatics Section, Information Technology Department, Faculty of Medicine, Ramathibodi Hospital, Mahidol University, Bangkok, Thailand; 4 Nephrology Division, Department of Medicine, Bhumibol Adulyadej Hospital, Bangkok, Thailand; 5 Center for Public Health, School of Medicine, Dentistry and Biomedical Sciences, Queen’s University Belfast, Northern Ireland, UK; 6 Centre for Clinical Epidemiology and Biostatistics, Hunter Medical Research Institute, School of Medicine and Public Health, University of Newcastle, New Lambton, NSW Australia

**Keywords:** End stage renal disease, Hyperphosphatemia, Phosphate binders, Real-world evidence, Treatment effect model

## Abstract

**Background:**

Uncontrolled hyperphosphatemia in end stage renal disease (ESRD) increases the risk of cardiovascular disease (CVD), bone disorders, and premature mortality. Randomized controlled trials show reduced CVD risk of non-calcium-based phosphate-binders (NCBPBs) compared to CBPBs although evidence from real world data is less consistent. This study aimed to compare the effectiveness of NCBPBs, CBPBs, to no phosphate-binder (PB) on mortality and cardiovascular disease in Thai hyperphosphatemic ESRDs.

**Methods:**

A retrospective-cohort was conducted by using data from 2 university hospitals between January 2010 and July 2020 (COA. MURA2020/1398 and IRB No.100/63). Primary outcomes were overall survival (OS) and CVD-free time. Secondary outcomes included bone disorders following ESRD. An inverse-probability weighting with regression adjustment was used to assess treatment effects.

**Results:**

A total of 8,005 patients were included. Initial CBPBs were associated with both longer OS and CVD-free time compared to no-PBs, while initial treatment with aluminum hydroxide was the highest risk of bone disorders. Patients who received CBPBs-NCBPBs had longest OS, followed by aluminum hydroxide, and CBPBs, with average OS of 13.5, 11.0, and 10.9 years, respectively. The average CVD-free time was longest for the CBPBs-NCBPBs, followed by CBPBs-CBPBs compared to no-PBs. However, these comparisons were insignificantly different.

**Conclusions:**

initial hyperphosphatemic ESRD treatment with CBPBs provided longer OS and CVD-free time compared to no-PBs, while aluminum hydroxide was the highest risk of bone disorders. CBPBs followed by NCBPBs achieved the longest OS and CVD-free time, although these were statistical non-significance.

**Supplementary Information:**

The online version contains supplementary material available at 10.1186/s12882-025-04058-7.

## Background

End-stage renal disease (ESRD) commonly leads to hyperphosphatemia, with estimates [1–6]; in turn, untreated hyperphosphatemia leads to increased risk of bone disorders (such as osteoporosis or fractures), secondary hyperparathyroidism, vascular calcification, cardiovascular events (CVD) and premature mortality [7–13]. Clinical practice guidelines [10, 14, 15] recommend the use of phosphate binders (PBs) for ESRD patients where serum phosphate levels exceed 4.5 mg/dl. Calcium-based PBs (CBPBs) are the recommended primary treatment option [10, 14, 15], and if ineffective or contraindicated, non-calcium-based PBs (NCBPBs, such as sevelamer, lanthanum carbonate), or aluminum hydroxide can be considered as potential secondary treatments [10, 14, 15]. 

In real-world clinical practice, national PB prescribing practices differ between countries due to factors that include healthcare provision, affordability, accessibility, and availability of medications [16–18]. Many of these medications tend to be more commonly available, affordable, and reimbursable as a primary treatment option in high-income countries (HICs). In contrast, several of the more recently available NCBPBs are expensive (with the exception of aluminum hydroxide) and not widely funded in developing or upper-middle-income countries (UMICs), including Thailand. Only a minority of patients are able to afford sevelamer or lanthanum carbonate, and therefore aluminum hydroxide is more commonly used instead [14, 15]. 

Previous evidence from network meta-analyses (NMA) of randomized controlled trials (RCT) [19, 20] of chronic kidney disease (CKD) patients has shown increases in all-cause mortality (between 76% and 89%) in those treated with CBPBs compared to those treated with NCBPBs and sevelamer, although this did not reach statistical significance with lanthanum. In addition, CBPBs were also found to lead to increase serum calcium levels relative to sevelamer and lanthanum carbonate [20]. However, evidence relating to CVD events, ESRD-related bone disorders, and premature mortality are still lacking, especially with regards to sevelamer and lanthanum carbonate treatment outcomes [19–22]. Robust evaluation of evidence from non-RCT settings is still required to demonstrate clinical utility in real-world practice.

Previous evidence from non-RCT studies [23–26] adjusted for confounding using propensity score (PS) approaches was generally consistent with benefit. The largest cohort study [23] (*n* = 4,721) that followed up ESRD patients for 6 months demonstrated a 4% lower risk of CVD events and all-cause mortality associated with sevelamer compared to CBPBs, although this was not statistically significant. Similar findings were also reported in another PS-matched cohort (*n* = 3,176)^24^ that had a median follow-up time of 1.5 years. Other studies that used either matched [26] or unmatched PS [25] with follow-up times of 2 and 1.2 years respectively showed significantly lower mortality rates (ranging between 23 and 35%) associated with sevelamer and lanthanum treatments.

However, previous non-RCT studies [23–26] were conducted exclusively in HICs and solely focused on the initial treatment regardless of secondary treatment options, which may be susceptible to potential confounding. For instance, in Thailand and many other UMICs, NCBPBs, (with the exception of aluminum hydroxide) are considered an add-on or substitute option, rather than a primary treatment option. Furthermore, previous observational studies have not considered bone disorders associated with ESRD. As such, there is still insufficient evidence surrounding the use of PBs as primary and secondary treatment options to reduce the risk of CVD, bone disorders (including osteoporosis or fractures related to ESRD), and premature mortality in ESRD patients with hyperphosphatemia.

Therefore, this study aimed to evaluate the effectiveness of different PB classes (i.e., CBPBs, NCBPBs) to non-PBs on mortality and cardiovascular disease in Thai hyperphosphatemic ESRD patients.

## Methods

### Study design and setting

A multicenter retrospective cohort study was conducted using data from both the Ramathibodi and Bhumibol Adulyadej Hospitals in Bangkok, Thailand. All relevant patient data were retrieved from electronic healthcare records from the 1st January 2010 to 31st July 2020. The study protocol was approved by the Committee of Research, Faculty of Medicine Ramathibodi Hospital (COA. MURA2020/1398) and Bhumibol Adulyadej Hospital (IRB No.100/63).

### Study patients

Eligible patients were adults (> 18 years) with ESRD or with a persistent eGFR < 15 ml/min/1.73 m [2] on more than 2 consecutive occasions for more than 3 months, and hyperphosphatemia (serum phosphate > 4.5 mg/dl or 1.44 mmol/L) on 2 consecutive occasions for more than 3 months. Patients followed-up for 3 months or less were excluded.

### Treatments of interest

The treatments of interest were CBPBs, NCBPBs (i.e., lanthanum/or sevelamer), and aluminum hydroxide prescribed for at least 3 months for comparison with a control group that was not treated with a PB or subject to any dietary phosphate restriction [10, 14, 15]. Prescription of CBPBs and aluminum hydroxide were used to confirm hyperphosphatemia. In addition, time at administration of CBPBs during meals, obtained from electronic prescription databases, was used to verify indication of CBPBs for hyperphosphatemia. An index date (or baseline date) was defined as the date initiated PBs for PB groups, and date diagnosis of ESRD for no-PB group.

### Emulation of target trial

Three approaches were applied to emulate a target trial design to evaluate the various prescribing patterns in a clinical setting (see Supplementary Method S1 and Figure S1). First, an intention to treat (ITT) approach considered patient outcomes following initial treatments regardless of secondary treatments prescribed. Second, a per-protocol analysis (PPA) approach considered only patients who were prescribed PBs persistently until study-end (31st July 2020); patients who switched or were given secondary treatments were excluded. Third, clinical endpoints were considered specific to the actual treatment-patterns prescribed including CBPBs, NCBPBs (i.e., lanthanum/sevelamer) or aluminum hydroxide, when the first-line (i.e., CBPBs) was failed.

### Outcomes of interest

Primary outcomes were overall survival (OS) and time to CVD occurrence. OS was defined as the time taken from the date of treatment initiation to the date of death from any cause. Patients were censored if they were lost to follow up or still alive at study end. Mortality data (date and cause) was verified by death certificates obtained from the Division for Strategy and Planning, the Ministry of Public Health. Time to CVD was defined as the time from treatment initiation until the date on which a CVD event was recorded; CVD was defined as coronary heart disease (i.e., angina and myocardial infarction), heart failure, cerebrovascular or peripheral vascular disease [27, 28]. Secondary outcomes included reported bone disorders defined as the first occurrence of a bone disorder in patients with ESRD which included osteoporosis and/or any fracture (i.e., hip, wrist, vertebral, humerus, trochanteric, rib, pelvis, shoulder or arm, femur, ankle or feet).

### Covariables

Study covariables at baseline (i.e., initial treatments/diagnosis of hyperphosphatemia) and follow-up were retrieved from the electronic records (see supplementary Table S1) and included patient characteristics, comorbidities, renal replacement therapy (RRT) status (i.e., both hemodialysis (HD) and peritoneal dialysis (PD)), laboratory measurements, and additional medications prescribed. eGFR was estimated using the CKD-EPI equation [29]. Relevant medications were used to further verify ICD 9–10 codes for disease diagnosis.

For missing data, fixed variables such as sex and reimbursement were imputed using a carry-forward and carry-backward approach based on each patient. Diagnostic data were imputed using a carry-forward. Time-varying variables such as laboratory data (e.g., kidney function, lipid profiles, CBC) and physical examination data (e.g., weight, height, BMI), data were aggregated into 180-day intervals. This approach allowed using the most recent available values within the specified intervals. For medication supply data, prescription records were carried forward within 180-day intervals to account for medication supply gaps.

### Statistical analysis

Baseline characteristics were described by intervention groups using frequencies and percentages for categorical variables, and means (SD) or medians (interquartile ranges (IQR)) for continuous variables. These variables were compared between groups using Chi-square or Fisher’s exact test, analysis of variance (ANOVA) or quantile regression, where appropriate.

Kaplan-Meier (KM) curves were constructed to estimate median time to end-points of interest, and log-rank tests were applied to compare KM survival curves by treatment group. Parametric survival analysis was applied with appropriate survival distributions based on the lowest Akaike’s Information Criterion (AIC) to assess treatment effects with appropriate adjustment. Covariables identified with a p-value < 0.1 in univariate parametric analyses were also considered in a multivariate parametric model related to each of the intervention groups. Adjusted HRs with 95% CIs were estimated.

Treatment effects of phosphate binders (PBs) were estimated using a likelihood-adjusted-censoring with inverse-probability-weighted regression adjustment (LAC-IPWRA) approach, clearly described in detail in Supplementary Method S2. Briefly, this counterfactual approach involved: (1) creating treatment models using multi-logit regression to estimate the probability of receiving any PB, i.e., PS; (2) fitting parametric survival models weighted by the inverse of the estimated PS; and (3) assessing model assumptions including covariate balance (absolute standardized mean difference (SMD) < 0.20 and variance ratio ≈ 1) [30] and sufficient overlap in treatment probabilities [30, 31]. Potential-outcome means (POMs) as mean time to outcomes of interest and average treatment effects (ATE) were then estimated.

Sensitivity analysis was performed to evaluate the presence of unmeasured confounders by estimating an E-value [32, 33] (see Supplementary Method S3). All analyses were performed based on ITT, PPA, and actual treatment-pattern approaches using STATA 16.0. A two-sided p-value < 0.05 was considered statistically significant.

## Results

### Target patients

A total of 8,005 ESRD patients with hyperphosphatemia were included in the ITT approach, see supplementary Table S2. Baseline characteristics by treatment group are described in Table 1. A total of 28 covariates significantly differed between the four treatment groups, with the exception of reported comorbidities (such as cancer and hypoparathyroidism) and laboratory test data (i.e., HbA1C, LDL, HDL, iron, TIBC, and ferritin levels). These baseline characteristics are also described for participants included in the PPA and actual treatment-pattern approaches, see supplement Table S3-S4. Baseline covariates that differed significantly between treatment groups were further considered within the treatment model.

**Table 1:** Baseline characteristics of patients among treatment groups by ITT

Variables	*N*	% Missing	Treatment group				Total	*p*-value
			No-PBs	CBPBs	NCBPBs	Aluminum hydroxide		
**Total number of patients**,** n (%)**	8,005	0	3,736 (46.67)	4,071(50.86)	118 (1.47)	80 (1.00)	8,005 (100)	
**Hospital**,** n(%)**	8,005	0						< 0.001
Ramathibodi hospital			3,283 (87.87)	2,755 (67.67)	85 (72.03)	44 (55.00)	6,167 (77.04)	
Bhumibol hospital			453 (12.13)	1,316 (32.33)	33 (27.97)	36 (45.00)	1,838 (22.96)	
**Age (years)**,** mean(SD)**	8,005	0	48.50 (17.08)	61.39 (15.98)	61.16 (15.23)	53.15 (14.16)	55.29 (17.67)	< 0.001
**Male**,** n(%)**	8,005	0	2,172 (58.14)	1,937 (48.22)	65 (55.08)	41 (51.25)	4,215 (52.65)	< 0.001
**Reimbursement**,** n(%)**	8,005	0						< 0.001
Non-CSMBS			2,723 (72.89)	2,042 (50.16)	29 (24.58)	53 (66.25)	4,847 (60.55)	
CSMBS			1,013 (27.11)	2,029 (49.84)	89 (75.42)	27 (33.75)	3,158 (39.45)	
**BMI (kg/m**^**2**^**)**,** mean(SD)**	3432	57	24.15 (4.72)	24.96 (5.20)	26.09 (5.73)	25.13 (5.01)	2,4.51 (4.95)	< 0.001
**Marital status**,** n(%)**	8,005	0						< 0.001
Single			1,190 (31.85)	888 (21.81)	19 (16.10)	29 (36.25)	2,126 (26.56)	
Married			2,178 (58.30)	2,602 (63.92)	86 (72.88)	41 (51.25)	4,907 (61.30)	
Divorced/widowed			364 (9.74)	569 (13.98)	13 (11.02)	10 (12.50)	956 (11.94)	
Priest			0 (0.00)	2 (0.05)	0 (0.00)	0 (0.00)	2 (0.02)	
Unknown			4 (0.11)	10 (0.25)	0 (0.00)	0 (0.00)	14 (0.17)	
**Education levels**,** n(%)**	3,631	55						< 0.001
Primary level or less			589 (23.15)	310 (31.31)	12 (25.00)	7 (31.82)	918 (25.47)	
Secondary level			766 (30.11)	192 (19.39)	5 (10.42)	4 (18.18)	967 (26.83)	
University level or more			1,189 (46.74)	488 (49.29)	31 (64.58)	11 (50.00)	1,719 (47.70)	
**Renal replacement therapy**,** n(%)**	8,005	0						< 0.001
No RRT			2,333 (62.45)	3,048 (74.87)	47 (39.83)	48 (60.00)	5,476 (68.41)	
RRT			1,403 (37.55)	1,023 (25.13)	71 (60.17)	32 (40.00)	2,529 (31.59)	
**Comorbid**,** n(%)**								
Hypertension	8,005	0	1,874 (50.16)	3,940 (96.78)	98 (83.05)	73 (91.25)	5,985 (74.77)	< 0.001
Diabetes	8,005	0	871 (23.31)	2,152 (52.86)	48 (40.68)	33 (41.25)	3,104 (38.78)	< 0.001
Dyslipidemia	8,005	0	965 (25.83)	2,845 (69.88)	70 (59.32)	40 (50.00)	3,920 (48.97)	< 0.001
CVD	8,005	0	503 (13.46)	1,217 (29.89)	29 (24.58)	11 (13.75)	1,759(21.97)	< 0.001
Bone disorder related ESRD	8,005	0	78 (2.09)	448 (11.00)	4 (3.39)	2 (2.50)	533 (6.66)	< 0.001
Secondary hyperparathyroidism	8,005	0	314 (8.40)	1,195 (29.35)	61 (51.69)	32 (40.00)	1,602 (20.01)	< 0.001
Hypoparathyroidism	8,005	0	0 (0.00)	9 (0.22)	0 (0.00)	0 (0.00)	9 (0.11)	0.065
Gout	8,005	0	141 (3.77)	304 (7.47)	6 (5.08)	5 (6.25)	456 (5.70)	< 0.001
Liver disease	8,005	0	150 (4.01)	257 (6.31)	5 (4.24)	3 (3.75)	415 (5.18)	< 0.001
SLE	8,005	0	50 (1.34)	92 (2.26)	1 (0.85)	1 (1.25)	144 (1.80)	0.032
Cancer	8,005	0	27 (0.72)	39 (0.96)	1 (0.85)	0 (0.00)	67 (0.84)	0.630
AIDS	8,005	0	8 (0.21)	25 (0.61)	2 (1.69)	1 (1.25)	36 (0.45)	< 0.001
GI disorder	8,005	0	127 (3.40)	468 (11.50)	12 (10.17)	6 (7.50)	613 (7.66)	< 0.001
Chronic pulmonary disease	8,005	0	55 (1.47)	153 (3.76)	5 (4.24)	1 (1.25)	214 (2.67)	< 0.001
**Laboratory**,** median (IQR)**								
eGFR, ml/min/1.73 m^2^	7,983	0	5.0 (3.8, 8.0)	7.4 (4.6, 11.6)	5.6 (4.2, 8.8)	4.6 (3.4, 6.6)	6.0 (4.0, 10.2)	< 0.001
Phosphorus, mg/dL	8,005	0	5.6 (5.0, 6.6)	5.3 (4.8, 6.4)	5.8 (5.0, 7.0)	7.3 (6.1, 8.7)	5.4 (4.9, 6.6)	< 0.001
Corrected calcium, mg/dL	8,005	0	9.4 (8.8, 10.0)	9.4 (8.8, 9.9)	10.1 (9.4, 10.8)	10.1 (9.4, 10.8)	9.4 (8.8, 10.0)	< 0.001
PTH, pg/dL	4,608	42	319.6 (141.8, 639.2)	180.9 (87.0, 360.9)	382.8 (175.6, 1100.0)	463.0 (170.0, 800.2)	240.4 (109.0, 497.4)	< 0.001
Hemoglobin, g/dL	8,005	0	10.5 (9.1, 11.9)	9.8 (8.5, 11.1)	10.9 (9.3, 11.7)	9.9 (8.3, 11.3)	10.1 (8.8, 11.5)	< 0.001
Albumin, g/dL	8,005	0	3.6 (3.1, 4.0)	3.4 (2.9, 3.9)	3.5 (3.1, 3.9)	3.7 (3.0, 4.5)	3.5 (3.0, 3.9)	< 0.001
Uric, mg/dL	7,132	11	7.0 (5.6, 8.5)	7.8 (6.3, 9.4)	7.0 (5.2, 8.5)	7.3 (6.0, 8.8)	7.4 (5.9, 9.0)	< 0.001
HbA1C, mg%	2,981	63	6.3 (5.6, 7.3)	6.3 (5.6, 7.3)	6.0 (5.4, 7.3)	7.3 (6.0, 8.6)	6.3 (5.6, 7.3)	0.066
LDL, mg/dL	4,884	39	101.0 (79.0, 128.0)	103.4 (81.0, 132.4)	100.0 (79.0, 123.0)	105.5 (81.8, 140.0)	102.4 (80.0, 130.0)	0.066
Triglyceride, mg/dL	6,915	14	115.5 (82.0, 158.0)	128.0 (92.0, 183.0)	122.4 (90.2, 173.0)	139.0 (78.6, 186.0)	122.4 (87.0, 172.0)	< 0.001
HDL, mg/dL	4,684	41	45.0 (37.0, 56.0)	45.0 (37.0, 56.0)	47.0 (37.0, 55.0)	44.0 (38.0, 56.0)	45.0 (37.0, 56.0)	0.932
Iron, ug/dL	2,933	63	51.0 (34.5, 71.0)	53.0 (36.0, 73.0)	51.0 (37.0, 69.0)	55.5 (36.0, 82.0)	52.0 (36.0, 73.0)	0.252
TIBC, ug/dL	2,946	63	208.9 (172.6, 245.0)	207.9 (173.9, 247.0)	215.0 (181.0, 238.0)	203.0 (171.3, 233.0)	208.0 (173.0, 246.0)	0.899
Ferritin, ng/dL	482	94	188.0 (165.0, 217.0)	181.0 (157.0, 212.0)	183.0 (167.0, 237.0)	187.0 (150.0, 247.0)	182.0 (157.0, 213.0)	0.178

Abbreviations: Body mass index; (BMI), CBPBs; Calcium-based phosphate binders, CVD; Cardiovascular disease, CSMBS; Civil Servant Medical Benefit Scheme, eGFR; Estimated Glomerular Filtration Rate, ESRD; End-stage renal disease, HBA1C; Hemoglobin A1C, HDL; High-Density Lipoprotein, HIV; Human immunodeficiency virus, IQR; Interquartile range, ITT; Intention to treat, GI; Gastrointestinal tract, LDL; Low-Density Lipoprotein, NCBPBs; N; number, Non-calcium-based phosphate binders i.e., lanthanum or sevelamer; OS; Overall survival outcome, PBs; Phosphate binders, PPA; Per-protocol analysis, SLE; PTH; Parathyroid hormone levels, RRT, Renal Replacement Therapy, TIBC; Total iron binding capacity

### Overall survival

A total of 8,005 patients representing 35,355.8 person-years were included in the ITT approach with an overall median (IQR) follow-up time of 4.0 (2.0, 6.7) years; the corresponding median follow-up times for the no-PBs, CBPBs, NCBPBs, and aluminum hydroxide groups were 3.88, 4.14, 2.77, and 4.72 years. (see supplementary Table S5). Crude incidence rates [IRs (95% CI)] for all-cause mortality were 7.1 (6.7, 7.5), 10.3 (9.9, 10.8), 9.3 (6.8, 12.6), and 10.6 (7.9, 14.2)/100 person/years for no-PBs, CBPBs, NCBPBs, and aluminum hydroxide groups, respectively (see Table 2). KM curves by treatment approach were constructed (supplementary Figure S2) indicating median OS in aluminum hydroxide, CBPBs, and NCBPBs of 6.4, 6.6, and 8.2 years whereas a median OS in the no-PBs treatment was longer than 10 years. Crude IRs for all treatments based on the PPA and actual treatment-pattern approaches trended to be higher than those observed for the ITT analysis, with the exception of NCBPBs, and aluminum hydroxide, for which IRs tended to be lower in patients in receipt of secondary treatment, see Table 2.

**Table 2:** Estimated incident rates for all-cause mortality, cardiovascular events, and bone disorder-related to ESRD

Treatment approaches	Treatments	All-cause mortality		CVD		Bone disorder	
		No events (Person-year)	IR/100/ years	No events (Person-year)	IR/100/ years	No events (Person-year)	IR/100/ years
ITT	No-PBs	1,146 (16,193.7)	7.1 (6.7, 7.5)	820 (8,981.6)	9.1 (8.5, 9.8)	117 (15,240.8)	0.8 (0.6, 0.9)
	CBPBs	1,994 (19,293.5)	10.3 (9.9, 10.8)	1,211 (9,091.4)	13.3 (12.6, 14.1)	426 (15,949.3)	2.7 (2.4, 2.9)
	NCBPBs	41 (443.2)	9.2 (6.8, 12.6)	24 (232.9)	10.30 (6.9, 15.4)	12 (394.9)	3.0 (1.7, 55.4)
	Aluminum hydroxide	45 (425.4)	10.6 (7.9, 14.2)	27 (254.7)	10.6 (7.3, 15.5)	14 (367.9)	3.8 (2.3, 6.4)
	**Overall**	3,226 (36,355.1)	8.9 (8.6, 9.2)	2,082 (18,560.6)	11.2 (10.7, 11.7)	569 (31,952.9)	1.8 (1.6, 1.9)
PPA	No-PBs	1,146 (16,193.7)	7.1 (6.7, 7.5)	820 (8,981.6)	9.1 (8.5, 9.8)	117 (15,240.8)	0.8 (0.6, 0.9)
	CBPBs	1,619 (13,147.8)	12.3 (11.7, 12.9)	890 (6,518.2)	13.7 (12.7, 14.6)	287 (10,861.7)	2.6 (2.3, 2.9)
	NCBPBs	17 (139.3)	12.2 (7.6, 19.6)	10 (96.7)	10.34 (5.6, 19.2)	4 (131.7)	3.0 (1.1, 8.1)
	Aluminum hydroxide	4 (12.7)	31.5 (11.8, 83.9)	0 (3.6)	0	0 (12.7)	0
	**Overall**	2,748 (29,493.5)	9.8 (9.5, 10.2)	1,720 (15,600.14)	11.0 (10.5, 11.6)	408 (24,980.9)	1.6 (1.5, 1.8)
Actual treatment-patterns	No-PBs	1,146 (16,193.7)	7.1 (6.7, 7.5)	820 (8,981.6)	9.1 (8.5, 9.8)	117 (15,240.8)	0.8 (0.6, 0.9)
	CBPBs-CBPBs	1,749 (14,989.2)	11.7 (11.1, 12.2)	1,111 (7,928.4)	14.0 (13.2, 14.8)	347 (12,502.1)	2.8 (2.5, 3.1)
	CBPBs-NCBPBs	165 (2,758.7)	6.0 (5.1, 7.0)	57 (473.6)	12.1 (9.4, 15.7)	46 (2,135.4)	2.2 (1.6, 2.9)
	CBPBs-Aluminum	37 (626.1)	5.9 (4.3, 8.2)	16 (112.0)	14.3 (8.6, 23.3)	10 (542.6)	1.8 (1.0, 3.4)
	**Overall**	3,097 (34,567.7)	9.0 (8.6, 9.3)	1,990 (17,495.6)	11.4 (10.9, 11.9)	520 (30,420.9)	1.7 (1.6, 1.9)

Abbreviations: CBPBs; Calcium-based phosphate binders, CVD; Cardiovascular disease, CSMBS; IR; Incidence rate, ITT; Intention to treat, NCBPBs; N; Number, Non-calcium-based phosphate binders i.e., lanthanum or sevelamer; PBs; Phosphate binders, PPA; Per-protocol analysis

The OS model with Weibull regression and IPWRA for the ITT analysis indicated average OS times (95% CI) of 11.17 (9.75, 12.76), 11.45 (10.03, 12.87), 28.24 (-22.58, 79.06), and 7.26 (3.33, 11.18) years for no-PBs, CBPBs, NCBPBs, and aluminum hydroxide from time of initiation, respectively, see Table 3. For the PPA approach, aluminum hydroxide was omitted from analysis given the small number of patients (*n* = 7). Similar to the ITT approach, the estimated average OS times (95% CI) were 10.13 (9.14, 11.07), 10.68 (8.98, 11.59), and 12.38 (-27.21, 45.22) years, for persistent use of no-PBs, CBPBs, and NCBPBs, respectively over the duration of the study period (see Table 3). For the actual treatment-patterns, the CBPBs-NCBPBs demonstrated the longest average OS time, followed by CBPBs-aluminum, CBPBs-CBPBs, and no-PBs, with average times (95% CI) of 13.49 (10.03, 16.93), 11.01 (7.54, 14.48), 10.86 (8.46, 11.96), and 10.42 (9.94, 11.20), respectively (see Table 3), although none of these differed significantly.

**Table 3:** Estimated treatment effects on mean survival and CVD-free time and HRs by treatment group for all approaches: LAC-IPWRA and adjusted HRs by Weibull regression with PS adjustment

Treatments	OS		Time to CVD	
	Mean OS (LAC-IPWRA)	Adjusted HR (Conventional PS)	Mean CVD-free time (LAC-IPWRA)	Adjusted HR (Conventional PS)
**ITT approach**				
No-PBs	11.17 (9.57, 12.76)	1 (ref)	8.54 (7.28, 13.43)	1 (ref)
CBPBs	11.45 (10.03, 12.87)	0.96 (0.77, 0.99)	13.76 (10.93, 16.48)	0.95 (0.86, 1.05)
NCBPBs	28.24 (-22.58, 79.06)	0.87 (0.69, 1.30)	22.74 (-24.57, 99.04)	0.68 (0.45, 1.03)
Aluminum hydroxide	7.26 (3.33, 11.18)	1.08 (0.80, 1.45)	47.65 (-41.55, 136.85)	0.99 (0.67, 1.45)
**PPA approach**				
No-PBs	10.13 (9.14, 11.07)	1 (ref)	8.57 (7.89, 9.25)	1 (ref)
CBPBs	10.68 (8.98, 11.59)	0.90 (0.87, 1.08)	9.09 (7.75, 12.21)	0.97 (0.87, 1.08)
NCBPBs	12.38 (-27.21, 45.22)	0.86 (0.54, 1.46)	17.69 (-22.27, 79.65)	0.68 (0.36, 1.27)
Aluminum hydroxide	excluded	excluded	excluded	excluded
**Actual treatment-patterns approach**				
No-PBs	10.42 (9.94, 11.20)	1 (ref)	8.19 (5.67, 8.97)	1 (ref)
CBPBs-CBPBs	10.86 (8.46, 11.96)	0.91 (0.79, 1.01)	9.84 (6.18, 12.36)	0.84 (0.56, 1.08)
CBPBs-NCBPBs	13.49 (10.03, 16.93)	0.77 (0.58, 1.16)	11.27 (6.83, 15.91)	0.77 (0.45, 1.06)
CBPBs-Aluminum	11.01 (7.54, 14.48)	0.92 (0.70, 1.32)	75.96 (-50.61, 184.53)	1.39 (0.85, 2.25)

Abbreviations: CBPBs; Calcium-based phosphate binders, CVD; Cardiovascular disease, HR; Hazard ratio, ITT; Intention to treat, LAC-IPWRA; Likelihood-adjusted-censoring inverse-probability-weighted regression adjustment, NCBPBs; Non-calcium-based phosphate binders i.e., lanthanum or sevelamer, OS; Overall survival outcome, PBs; Phosphate binders, PPA; Per-protocol analysis, PS; Propensity score, Ref; Reference

### CVD events

Of 8,005 people in the entire cohort, the baseline characteristics, comorbidities, and laboratory data are described for 6,246 in the CVD cohort (see supplementary Table S3-S4).

The overall median (IQR) follow-up time was 2.1 (0.8, 4.5) years, with the no-PBs, CBPBs, NCBPBs, and aluminum hydroxide groups having median follow-up times of 1.98, 2.22, 2.07, and 2.06 years, respectively. Crude IRs (95%CI) for CVD events were 9.1 (8.5, 9.8), 13.3 (12.6, 14.1), 10.3 (6.9, 15.4), and 10.6 (7.3, 15.5)/100 person/years for no-PBs, CBPBs, NCBPBs, and aluminum hydroxide groups, respectively (see Table 2). For the ITT approach, the median times to a CVD event for CBPBs and aluminum hydroxide groups were 5.8 and 10.0 years, while these median times were longer than 10 years for the remaining treatment groups, see supplementary Figure S3. Crude IRs for CVD based on the PPA and actual treatment-pattern approaches trended to be higher compared to those for the ITT analysis for all treatments, except for aluminum hydroxide, where no CVD events were recorded, given the small number of patients (see Table 2 and supplementary Figure S3).

The IPWRA model by ITT suggested that the mean times for patients to remain free of a recorded CVD event (95% CI) were 8.54 (7.28, 13.43), 13.76 (10.93, 16.48), 22.74 (-24.57, 99.04), and 47.65 (-41.55, 136.85) years for no-PBs, CBPBs, NCBPB, and aluminum hydroxide respectively (see Table 3).

### Bone disorders

After adjusting for PS using a Weibull survival approach (see supplementary Table S6), CBPBs, NCBPBs, and aluminum hydroxide showed a 2.51 (2.01, 3.15), 2.02 (1.10, 3.71), and 3.94 (2.24, 6.91) times higher risk of bone disorder presentation than no-PBs.

### Sensitivity analysis

A sensitivity analysis to assessed unmeasured confounding through estimated E-values (see Supplementary Table S7) indicated an E-value of CBPBs-NCBPBs versus CBPBs-CBPBs of 4.45, i.e., the mean difference in OS between CBPBs-NCBPBs and CBPBs-CBPBs could be explained by unmeasured confounder associated with both treatment and outcome if an effect was *≥* 4.45-fold; residuals with lower levels of confounding would therefore not explain the effect observed. For comparisons between CBPBs-NCBPBs and no-PBs, an E-value estimate of 5.74 indicated an unmeasured confounder with an effect *≥* 5.7 would be required to explain the effect observed. Thus, the evidence for causal associations between treatments and OS (i.e., CBPBs-NCBPBs versus CBPBs-CBPBs, and CBPBs-NCBPBs versus no-PBs) appears reasonably strong.

A sensitivity analysis was also performed by recalculating the PS by including the comorbidities (DM, HT, DLP, secondary hyperparathyroidism) initially excluded from the PS model due to model non-convergence (HT) and violations of conditional independence assumptions and treatment probability overlap (DM, DLP, hyperparathyroidism). Results were consistent with the primary analysis, except for increased uncertainty in mean survival time estimates for NCBP treatment in ITT (28.24 [-22.58, 79.06] vs. 40.12 [-16.34, 96.59]) and PPA (12.38 [-27.21, 45.22] vs. 109.52 [-274.19, 493.22]) analyses. (See Supplementary Method S3).

## Discussion

We conducted a retrospective cohort of real-world practice data by including 8,005 ESRD patients with hyperphosphatemia. A target trial was emulated to determine which initial PBs and treatment options were most effective for ESRD patients with hyperphosphatemia. The ITT approach with IPWRA indicated that NCBPBs provided the longest OS and CVD event-free time, 28 and 12 years respectively in comparison to 11 and 10 years for CBPBs relative to patients treated with no-PBs. However, both NCBPBs and CBPBs were associated with a 2.5 and 2 fold greater risk of bone disorders.

Only 2.5% of all ESRD patients received NCBPBs or aluminum hydroxide treatments initially, with 14.8% initially receiving CBPBs. Those patients who were switched to or also received NCBPBs in addition to their primary treatment, had the longest OS and CVD-free time; however, these findings were imprecise and not statistically significant due to a small number of patients who received NCBPBs.

Previous studies that focused solely on initial treatment with PBs [19, 20, 23–26], did not consider the effects in those patients that switched to or additionally received NCBPBs. Furthermore, CVD and bone disorders outcomes were also not considered [23, 34–36]. More recent NMA evidence from RCTs [19, 20] suggested that CBPBs increased all-cause mortality compared to NCBPBs, particularly for lanthanum and sevelamer. CBPBs may reduce mortality risk compared to diet restriction, but not significantly [19, 20]. Their findings supported our conclusion that initial CBPBs were beneficial compared to no-PBs, but not significant. Our findings for the initial use of NCBPBs as a treatment option appears to offer longer OS and CVD event-free time compared to CBPBs; these findings are consistent with previous non-RCTs that used PS with IPTW [23] and PS matching [24]. In contrast, our findings were inconsistent with a conventional PS study [25] that adopted an ITT approach which suggested initial treatment with sevelamer significantly reduced all-cause mortality compared to CBPB treatments. As such, the reported effects associated with initial NCBPB treatment options, remain inconsistent.

### Comparison of treatment approaches

Different treatment approaches were adopted to reduce the bias associated with real-world data. While an ITT approach minimizes bias in RCTs, it may not sufficiently represent changing clinical treatment options in real-world practice given these are dependent on the changing condition of the patient and further limited by the economic constraints of healthcare provision. Additional approaches that consider PPA and actual treatment-pattern interventions may provide more robust comparisons that capture the versatility of clinical decision making and reduce potential ambiguities associated with misclassification or issues of causal inference errors. However, such approaches may also be more prone to selection bias through the exclusion of certain patients for clinical or non-clinical reasons. Given that some patients may receive the primary treatment option for only a relatively short period of time, an ITT approach may limit the evaluation of the long-term clinical outcomes for that treatment option. As such, evaluation approaches that also consider secondary treatment options may be more appropriate in real-world practice settings as many most patients initially prescribed CBPBs may switch to or also receive additional NCBPBs (i.e., lanthanum/sevelamer), particularly if CBPBs show insufficient efficacy or lead to side effects. Nevertheless, many healthcare providers, particularly in middle- or upper-income countries have limited accessibility to NCBPBs as a primary treatment option.

### Strengths and limitations

This study had several strengths. First, 8,005 ESRD patients with hyperphosphatemia were included, which is sizeable given the scale of previous studies. Second, this study evaluated all available PBs, and focused on all important clinically relevant outcomes including death, CVD events, and bone disorders. Third, this study emulated a target trial based on a cohort from real-world practice. Lastly, this study extended previous research by using a counterfactual approach to ascertain which initial PBs and treatment options are the most effective in a real-world setting. Counterfactual approaches reduce the bias associated with observational effect estimates by considering confounders that affect both outcomes and treatment selection in order to improve the comparability between treatment groups. While some comorbidities (DM, HT, DLP, secondary hyperparathyroidism) were excluded from the PS calculation, they were included in the outcome models. Sensitivity analyses also confirmed the robustness of our findings.

Notwithstanding, this study had several limitations. First, the small number of patients initially prescribed NCBPBs necessitated combining lanthanum and sevelamer, but still leading to imprecise treatment effect estimates and precluding subgroup analyses (by hypercalcemia or dialysis modality). Second, we could not directly assess drug adherence although it was indirectly assessed from the number of PB prescriptions identified from the billing database. Third, the different defining the index date (diagnosis date for no-PB, treatment initiation date for PB groups) introduces the potential for lead-time bias, particularly for the NCBPB group; this bias cannot be fully excluded. Fourth, the relatively short follow-up period (approximately 4 years for OS and CVD) and the shorter follow-up time observed in the NCBPB group (approximately 1–2 years shorter for OS) compared to other groups may influence the results. Longer follow-up in future studies is needed to mitigate potential survival bias. Fifth, the analysis considered only clinically reported variables. We lacked information on factors such as dietary intake, vascular calcification, bone mineral density, nutrition, and socio-demographic variables. Nevertheless, the sensitivity analysis using E-values was reassuring in that our findings were unlikely to be biased by unknown variables.

Further observational cohorts with a larger sample size might enable a more robust evaluation of individual NCBPBs, particularly lanthanum and sevelamer. Subgroup analyses by hypercalcemia or dialysis would also provide more accurate treatment effects associated with NCBPBs. Counterfactual prediction modelling would enable differentiation of patients who may derive additional benefit from the prescription of CBPBs or NCBPBs.

## Conclusion

Our findings suggest that patients with ESRD-hyperphosphatemia that receive CBPBs as an initial treatment option are more likely to benefit from longer OS and reduced CVD risk compared to patients that do not receive PBs. In addition, patients treated initially with aluminum hydroxide were at highest risk of bone disorders. The treatment option that included CBPBs initially followed by a secondary intervention of NCBPBs provided the best OS and lowest CVD risk compared to patients that were not treated with PBs. Nevertheless, none of our findings reach statistical significance and larger populations are needed.

## Electronic supplementary material

Below is the link to the electronic supplementary material.

Supplementary Material 1

## Data Availability

The data underlying this article will be shared on reasonable request to the corresponding author.

## Abbreviations

AIC: Akaike’s Information Criterion
ANOVA: Analysis of variance
ATE: Average treatment effects
CBPBs: Calcium-based phosphate-binders
CKD: Chronic kidney disease
CVD: Cardiovascular disease
ESRD: End stage renal disease
HICs: High-income countries
IQR: Interquartile ranges
ITT: Intention to treat
KM: Kaplan-Meier
LAC-IPWRA: Likelihood-adjusted-censoring with inverse-probability-weighted regression adjustment
NCBPBs: Non-calcium-based phosphate-binders
NMA: Network meta-analyses
OS: Overall survival
PB: Phosphate binders
PPA: Per-protocol analysis
PS: Propensity score
RCT: Randomized controlled trials
RRT: Renal replacement therapy
SMD: Standardized mean difference
UMICs: Upper-middle-income countries
