# Supplementary material for: Effectiveness of phosphate binders on mortality and cardiovascular disease in end-stage renal disease patients with hyperphosphatemia: a multicenter real-world cohort study
Source: BMC Nephrol. 2025 Mar 10;26:131. doi: 10.1186/s12882-025-04058-7 (PMC11895186; doi:10.1186/s12882-025-04058-7)
Supplement: Supplementary file 1 — Supplementary Material 1 [file 12882_2025_4058_MOESM1_ESM.docx]

**Supplementary materials**

**CLINICAL EFFECTIVENESS OF PHOSPHATE BINDERS FOR TREATING HYPERPHOSPHATEMIA IN END-STAGE RENAL DISEASE PATIENTS: A MULTICENTER REAL-WORLD STUDY**

# Supplement legends

| **Table S1.** | List of study covariables | *Page 2* |
| --- | --- | --- |
| **Table S2.** | Number of target patients by treatment approach | *Page 5* |
| **Table S3.** | Baseline characteristics of patients included in the PPA by treatments approach | *Page 6* |
| **Table S4.** | Baseline characteristics of patients included in the actual treatment-pattern analysis by treatment approach | Page 15 |
| **Table S5.** | Median (IQR) follow-up time by treatment approach and outcomes | *Page 24* |
| **Table S6.** | Estimation of PB effects on bone disorder: Parametric survival analysis with Weibull survival distribution (ITT approach) | *Page 25* |
| **Table S7.** | Summary E-value point estimates and confidence intervals by treatment approach: actual treatment-pattern analysis | *Page 26* |
| **Figure S1.** | Treatment approaches considered | *Page 27* |
| **Figure S2.** | KM curves for all-cause mortality by treatment approach | *Page 28* |
| **Figure S3.** | KM curves for CVD events by treatment approaches | *Page 29* |
| **Methods S1.** | Clinical trial emulation and treatment approaches | *Page 30* |
| **Methods S2.** | Statistical analysis | *Page 32* |
| **Methods S3.** | Sensitivity analysis | *Page 45* |

# Table S1*.* List of study covariates

| **Demographic variables** | **Variable name** | **Type/format** |
| --- | --- | --- |
| Age (year) | age | number |
| Sex | sex | male/female |
| Race | race | text |
| Province | province | text |
| Education (highest) | education | text |
| Marital status | mstatus | text |
| Height (cm) | ht | number |
| Body weight (kg) | Bw | number |
| Boda mass index (BMI) (kg/m^2^) | bmi | number |
| Reimbursement type | reimburse | text |
| Renal replacement therapy (RRT) initiation date | drrtini | DDMMMYYYY |
| RRT Access date | daccess | DDMMMYYYY |
| Type of RRT | modeRRT | No(0)/  Peritoneal dialysis (PD)(1)/  Hemodialysis (HD) (2)/  Kidney transplant (KT) (3) |
| **Diagnostic variables** | **ICD-9** | **ICD-10** |
| *ICD of target ESRD patients* | | |
| End stage renal disease | - | N180 |
| Chronic kidney disease, stage 5 | - | N185 |
| End stage renal disease (require dialysis) | - | N186 |
| Chronic kidney disease, unspecified | - | N189 |
| Encounter for fitting and adjustment of extracorporeal dialysis catheter | - | Z4901 |
| Encounter for fitting and adjustment of peritoneal dialysis catheter | - | Z4902 |
| Breakdown (mechanical) of intraperitoneal dialysis catheter | - | T85611 |
| Displacement of intraperitoneal dialysis catheter | - | T85621 |
| Leakage of intraperitoneal dialysis catheter | - | T85631 |
| Other mechanical complication of intraperitoneal dialysis catheter | - | T85691 |
| Infection and inflammatory reaction due to peritoneal dialysis catheter | - | T8571 |
| Arteriovenous fistula, acquired | - | I770 |
| Mechanical complication of vascular dialysis catheter | - | T824 |
| Mechanical complication of other cardiac and vascular devices and implants | - | T825 |
| Infection and inflammatory reaction due to other cardiac and vascular devices, implants and grafts | - | T827 |
| **Diagnostic variables** | **ICD-9** | **ICD-10** |
| Other specified complications of cardiac and vascular prosthetic devices, implants and grafts | - | T828 |
| Unspecified complication of cardiac and vascular prosthetic device, implant and graft | - | T829 |
| Kidney Transplant status | - | Z940 |
| Venous catheterization for renal dialysis | 3895 | - |
| Arteriovenostomy for renal dialysis | 3927 | - |
| Revision of arteriovenous shunt for renal dialysis | 3942 | - |
| Removal of arteriovenous shunt for renal dialysis | 3943 | - |
| Hemodialysis | 3995 | - |
| Creation of cutaneoperitoneal fistula | 5493 | - |
| Peritoneal dialysis | 5498 | - |
| Kidney transplantation | 5569 | - |
| *ICD of comorbidities* | | |
| Diabetes (DM) | - | E10 - E149 |
| Hypertension (HT) | - | I10 - I159 |
| Dyslipidemia | - | E780 - E789 |
| Obesity | - | E660 - E669 |
| Heart diseases | 3601-3607, 3610-3617, 3619, 4109 | I20, I200 - I259, I42, I420-I509 |
| Cerebrovascular accident (CVA)/stroke | - | I60 - I698 |
| Peripheral vascular disease (PVD) | - | I70 - I798 |
| Osteoporosis | - | M80 - M85 |
| Fractures | 7812-7815, 7852-7855, 7901-7905, 7911-7915, 7921-7925, 7932-7935, 7941,7942, 7945, 7951, 7952, 7955, 7973, 7975, 7976, 7983, 7985, 7986, 8153, 8155, 8171, 8172, 8174, 8175, 8151, 8152 | S12-S1291, S22-S2291, S32-S3281, S42-S4291, S52-S5291, S62-S6281, S72-S7291, S82-S8291, S92-S9291 |
| Liver disease  (cirrhosis, chronic hepatitis B, hepatitis C) | - | B15 - B19, K70 - K77 |
| Chronic pulmonary disease | - | J44 - J449, J45 - J46 |
| Any Cancer | - | C00 - C99 |
| Acquired Immunodeficiency Syndrome (AIDS) | - | B20 - B24 |
| Systemic Lupus Erythematosus (SLE) | - | M32 |
| Gout | - | M10 |
| Stone | - | N20 - N23 |
| Dementia | - | F00 - F03 |
| Any Handicapped | - | G80 - G83 |
| Pregnancy | - | O00 - O99, Z33 |
| Sleep disorder | - | G47, F51 |
| Periodontal disorder | - | K04 - K05 |
| GI disorders | - | K20-K31 |
| **Standard Medication group** | | |
| Phosphodiesterase inhibitors | Angiotensin-converting enzyme inhibitors (ACEIs)/Aldosterone receptor blockers (ARBs) | Sodium-Glucose Co-Transporter 2 (SGLT2) inhibitors, Dipeptidyl peptidase-4 (DPP-4) inhibitors |
| Glycoprotein IIB/IIIA inhibitors | P2Y12 receptor blockers | Thiazolidinedione (TZD) |
| Calcium-channel blockers | Beta-adrenergic antagonists | Sulfonylureas (SU) |
| Diuretics, Erythropoietin Stimulating Agent (ESA) | Alpha-1 receptor antagonist | Glucagon-like peptide 1 (GLP-1) receptor agonists |
| Vasodilators | Uric lowering agents,  Anti-dyslipidemia agents | Metformin, insulin, and other antidiabetic agents |
| Anticoagulants | Cyclo-oxygenase inhibitors (COX) inhibitors | Bisphosphonates, Vitamin D |
| Antiarrhythmic agents | Antiparathyroid agents | Heparins |
| **Laboratory variables** | **Unit** | **Lab code** |
| Serum creatinine (sCr) | mg/dL | 200058 |
| estimated Glomerular Filtration Rate (eGFR) | ml/min | 200718 |
| Albumin | g/dL | 200008 |
| Phosphorus | mg/dL | 200090 |
| serum intact parathyroid hormone (iPTH) | pg/dL | 200131 |
| Calcium (corrected) | mg/dL | calcium + 0.8 (4.0 - albumin) |
| Hemoglobin (Hb) | g/dL | 250001 |
| Hematocrit (Hct) | % | 250002, 250003 |
| Sodium | mEq/L | 200140 |
| Potassium | mEq/L | 200122 |
| Chloride | mEq/L | 200039 |
| Bicarbonate (HCO_3_) | mEq/L | 200037 |
| Fasting Blood Sugar (FBS) | mg/dL | 200076 |
| HbA1C | mg% | 100067 |
| Cholesterol | mg/dL | 200047 |
| Triglyceride | mg/dL | 200157 |
| Low-Density Lipoprotein (LDL) | mg/dL | 200106 |
| High-Density Lipoprotein (HDL) | mg/dL | 200085 |
| Uric acid | mg/dL | 200162 |
| Serum Iron | ug/dL | 200134 |
| Ferritin | ng/dL | 100072 |
| Total iron-binding capacity (TIBC) | Ug/dL | 100065 |
| Transferrin saturation (TSAT) | % | iron/transferrin * 71.24 |
| Hepatitis B surface antigen (HbsAg) | - | 100027 |
| Hepatitis B surface antibody (HbsAb) | - | 100028 |
| Antibody to hepatitis C virus (AntiHCV) | - | 100048 |
| Antibody to HIV virus (AntiHIV) | - | 100034, 100035, 100036 |

# Table S2*.* Number of target patients by treatment approach

| **Approaches** | **Treatments** | **Number of patients (%)** | | |
| --- | --- | --- | --- | --- |
|  |  | **OS** | **CVD** | **bone disorders** |
| ITT | No-PBs | 3,736 (46.67) | 3,233 (51.76) | 3,658 (48.96) |
|  | CBPBs | 4,071 (50.86) | 2,854 (45.69) | 3,623 (48.49) |
|  | NCBPBs | 118 (1.47) | 90 (1.53) | 113 (1.51) |
|  | Aluminum hydroxide | 80 (1.00) | 69 (1.10) | 78 (1.04) |
|  | **Total** | 8,005 (100) | 6,246 (100) | 7,472 (100) |
| PPA | No-PBs | 3,736 (53.52) | 3,233 (59.03) | 3,658 (55.95) |
|  | CBPBs | 3,184 (45.61) | 2,193 (40.04) | 2,821 (43.15) |
|  | NCBPBs | 54 (0.77) | 46 (0.84) | 52 (0.80) |
|  | Aluminum hydroxide | 7 (0.10) | 5 (0.09) | 7 (0.11) |
|  | **Total** | 6,981 (100) | 5,477 (100) | 6,538 (100) |
| Actual treatment-pattern | No-PBs | 3,736 (48.63) | 3,233 (53.82) | 3,658 (51.04) |
|  | CBPBs-CBPBs | 3,361 (43.75) | 2,449 (40.77) | 3,018 (42.11) |
|  | CBPBs-NCBPBs | 487 (6.34) | 240 (4.00) | 400 (5.58) |
|  | CBPBs-aluminum | 98 (1.28) | 85 (1.42) | 91 (1.27) |
|  | **Total** | 7,682 (100) | 6,007 (100) | 7,167 (100) |

Abbreviations: CBPBs; Calcium-based phosphate binders, CVD; cardiovascular disease, ITT; Intention to treat, OS; overall survival outcome, PBs; Phosphate binders, PPA; Per-protocol analysis,

# Table S3*.* Baseline characteristics of patients included in the PPA by treatments approach

| **Outcomes** | **Variables** | **N** | **% Missing** | **Treatment group** | | | | **Total** | **p-value** |
| --- | --- | --- | --- | --- | --- | --- | --- | --- | --- |
|  |  |  |  | **No-PBs** | **CBPBs** | **NCBPBs** | **Aluminum**  **hydroxide** |  |  |
| **OS** | **Total number of patients, n (%)** |  |  | 3,736 (53.5) | 3,184 (45.6) | 54 (0.8) | 7 (0.1) | 6,981 (100) |  |
|  | **Hospital, n(%)** | 6,981 | 0 |  |  |  |  |  | <0.001 |
|  | Ramathibodi Hospital |  |  | 3,283 (87.87) | 2,192 (68.84) | 37 (68.52) | 4 (57.14) | 5,516 (79.01) |  |
|  | Bhumibol Hospital |  |  | 453 (12.13) | 992 (31.16) | 17 (31.48) | 3 (42.86) | 1,465 (20.99) |  |
|  | **age (years), mean(SD)** | 6,981 | 0 | 48.50 (17.08) | 62.02 (16.09) | 63.18 (14.64) | 53.11 (16.60) | 54.78 (17.93) | <0.001 |
|  | **Female, n(%)** | 6,981 | 0 | 1,564 (41.86) | 1,696 (53.27) | 23 (42.59) | 4 (57.14) | 3,287 (47.08) | <0.001 |
|  | **Reimbursement, n(%)** | 6,981 | 0 |  |  |  |  |  | <0.001 |
|  | non-CSMBS |  |  | 2,723 (72.89) | 1,752 (55.03) | 12 (22.22) | 6 (85.71) | 4,493 (64.36) |  |
|  | CSMBS |  |  | 1,013 (27.11) | 1,432 (44.97) | 42 (77.78) | 1 (14.29) | 2,488 (35.64) |  |
|  | **BMI (kg/m^2^ ), mean(SD)** | 3,053 | 56 | 24.15 (4.72) | 24.95 (5.34) | 24.24 (5.45) | 23.35 (2.19) | 24.42 (4.96) | 0.001 |
|  | **Marital status, n(%)** | 6,981 | 0 |  |  |  |  |  | <0.001 |
|  | Single |  |  | 1,190 (31.85) | 705 (22.14) | 9 (16.67) | 4 (57.14) | 1,908 (27.33) |  |
|  | Married |  |  | 2,178 (58.30) | 2,012 (63.19) | 37 (68.52) | 3 (42.86) | 4,230 (60.59) |  |
|  | Divorced/widowed |  |  | 364 (9.74) | 456 (14.32) | 8 (14.81) | 0 (0.00) | 828 (11.86) |  |
|  | Priest |  |  | 0 (0.00) | 2 (0.06) | 0 (0.00) | 0 (0.00) | 2 (0.03) |  |
|  | Unknown |  |  | 4 (0.11) | 9 (0.28) | 0 (0.00) | 0 (0.00) | 13 (0.19) |  |
|  | **Education levels, n(%)** | 3,368 | 52 |  |  |  |  |  | <0.001 |
|  | Primary level or less |  |  | 589 (23.15) | 256 (32.04) | 6 (25.00) | 0 (0.00) | 851 (25.27) |  |
|  | Secondary level |  |  | 766 (30.11) | 166 (20.78) | 3 (12.50) | 0 (0.00) | 935 (27.76) |  |
|  | University level or more |  |  | 1,189 (46.74) | 377 (47.18) | 15 (62.50) | 1 (100.00) | 1,582 (46.97) |  |
|  | **Renal replacement therapy, n(%)** | 6,981 | 0 |  |  |  |  |  | <0.001 |
|  | No RRT |  |  | 2,333 (62.45) | 2,403 (75.47) | 25 (46.30) | 2 (28.57) | 4,763 (68.23) |  |
|  | RRT |  |  | 1,403 (37.55) | 781 (24.53) | 29 (53.70) | 5 (71.43) | 2,218 (31.77) |  |
|  | **Comorbid, n(%)** |  |  |  |  |  |  |  |  |
|  | Hypertension | 6,981 | 0 | 1,874 (50.16) | 3,077 (96.64) | 44 (81.48) | 7 (100.00) | 5,002 (71.65) | <0.001 |
|  | Diabetes | 6,981 | 0 | 871 (23.31) | 1,722 (54.08) | 20 (37.04) | 3 (42.86) | 2,616 (37.47) | <0.001 |
|  | Dyslipidemia | 6,981 | 0 | 965 (25.83) | 2,240 (70.35) | 32 (59.26) | 4 (57.14) | 3,241 (46.43) | <0.001 |

**Table S3**. Baseline characteristics of patients included in the PPA by treatments approach (cont.)

| **Outcomes** | **Variables** | **N** | **% Missing** | **Treatment group** | | | | | **Total** | **p-value** | |
| --- | --- | --- | --- | --- | --- | --- | --- | --- | --- | --- | --- |
|  |  |  |  | **No-PBs** | **CBPBs** | **NCBPBs** | **Aluminum**  **hydroxide** |  | |  |  |
| OS | CVD | 6,981 | 0 | 503 (13.46) | 991 (31.12) | 13 (24.07) | 2 (28.57) | 1,504 (21.54) | | <0.001 |  |
|  | Bone disorder related ESRD | 6,981 | 0 | 78 (2.09) | 363 (11.40) | 2 (3.70) | 0 (0.00) | 443 (6.35) | | <0.001 |  |
|  | Gout | 6,981 | 0 | 141 (3.77) | 243 (7.63) | 1 (1.85) | 0 (0.00) | 385 (5.51) | | <0.001 |  |
|  | Liver disease | 6,981 | 0 | 150 (4.01) | 204 (6.41) | 3 (5.56) | 0 (0.00) | 357 (5.11) | | <0.001 |  |
|  | SLE | 6,981 | 0 | 50 (1.34) | 77 (2.42) | 1 (1.85) | 0 (0.00) | 128 (1.83) | | 0.015 |  |
|  | Cancer | 6,981 | 0 | 27 (0.72) | 36 (1.13) | 1 (1.85) | 0 (0.00) | 64 (0.92) | | 0.217 |  |
|  | AIDS | 6,981 | 0 | 8 (0.21) | 22 (0.69) | 1 (1.85) | 0 (0.00) | 31 (0.44) | | 0.004 |  |
|  | GI disorder | 6,981 | 0 | 127 (3.40) | 373 (11.71) | 5 (9.26) | 2 (28.57) | 507 (7.26) | | <0.001 |  |
|  | Chronic pulmonary disease | 6,981 | 0 | 55 (1.47) | 122 (3.83) | 2 (3.70) | 0 (0.00) | 179 (2.56) | | <0.001 |  |
|  | Tumor lysis syndrome | 6,981 | 0 | 2 (0.05) | 0 (0.00) | 0 (0.00) | 0 (0.00) | 2 (0.03) | | 0.784 |  |
|  | Hypoparathyroidism | 6,981 | 0 | 0 (0.00) | 7 (0.22) | 0 (0.00) | 0 (0.00) | 7 (0.10) | | 0.079 |  |
|  | Secondary hyperparathyroidism | 6,981 | 0 | 314 (8.40) | 924 (29.02) | 32 (59.26) | 2 (28.57) | 1,272 (18.22) | | <0.001 |  |
|  | **Laboratory, median (IQR)** |  |  |  |  |  |  |  | |  |  |
|  | eGFR, ml/min/1.73 m^2^ | 6,963 | 0 | 5.0 (3.8, 8.0) | 7.8 (4.8, 12.0) | 5.9 (4.2, 9.4) | 4.6 (3.4, 11.4) | 6.0 (4.0, 10.4) | | <0.001 |  |
|  | Phosphorus, mg/dL | 6,981 | 0 | 5.6 (5.0, 6.6) | 5.1 (4.8, 6.2) | 5.6 (5.0, 6.9) | 6.2 (5.0, 8.1) | 5.4 (4.8, 6.6) | | <0.001 |  |
|  | PTH, pg/dL | 3,924 | 44 | 319.6  (141.8, 639.2) | 165.8  (79.9, 330.5) | 328.5  (150.6, 861.2) | 406.9  (44.0, 1304.4) | 237.8  (105.8, 499.0) | | <0.001 |  |
|  | Calcium, mg/dL | 6,981 | 0 | 9.4 (8.8, 10.0) | 9.4 (8.8, 9.9) | 10.0 (9.6, 10.6) | 10.4 (9.9, 11.8) | 9.4 (8.8, 10.0) | | <0.001 |  |
|  | Hemoglobin, g/dL | 6,981 | 0 | 10.5 (9.1, 11.9) | 9.7 (8.4, 11.1) | 10.8 (9.3, 11.7) | 9.6 (8.7, 11.5) | 10.2 (8.8, 11.5) | | <0.001 |  |
|  | Hematocrit, % | 1,896 | 73 | 29.0  (25.0, 33.0) | 28.9  (24.7, 33.0) | 30.6  (29.0, 34.7) | 31.4  (17.3, 38.0) | 29.0  (24.9, 33.0) | | 0.246 |  |
|  | Albumin, g/dL | 6,981 | 0 | 3.6 (3.1, 4.0) | 3.4 (2.8, 3.8) | 3.6 (3.3, 3.9) | 3.3 (3.2, 3.4) | 3.5 (3.0, 3.9) | | <0.001 |  |
|  | Sodium, mEq/L | 6,932 | 1 | 138.0  (136.0, 140.0) | 138.0  (134.0, 140.0) | 139.0  (136.0, 141.0) | 138.0  (136.0, 140.0) | 138.0  (135.0, 140.0) | | <0.001 |  |
|  | Potassium, mEq/L | 3,470 | 50 | 4.6 (4.0, 5.0) | 4.5 (4.0, 5.0) | 4.4 (4.0, 5.0) | 4.4 (3.8, 4.8) | 4.6 (4.0, 5.0) | | 0.144 |  |

**Table S3**. Baseline characteristics of patients included in the PPA by treatments approach (cont.)

| **Outcomes** | **Variables** | **N** | **% Missing** | **Treatment group** | | | | **Total** | **p-value** |
| --- | --- | --- | --- | --- | --- | --- | --- | --- | --- |
|  |  |  |  | **No-PBs** | **CBPBs** | **NCBPBs** | **Aluminum**  **hydroxide** |  |  |
| OS | Chloride, mEq/L | 6,938 | 1 | 100.0  (97.0, 104.0) | 103.0  (99.0, 107.0) | 100.5  (97.0, 104.0) | 100.0  (98.0, 105.0) | 101.0  (98.0, 105.0) | <0.001 |
|  | Carbondioxide, mEq/L | 6,943 | 1 | 22.8  (20.0, 25.5) | 21.0  (18.0, 24.4) | 21.1  (19.0, 24.2) | 22.0  (16.0, 27.5) | 22.0  (19.0, 25.0) | <0.001 |
|  | Uric, mg/dL | 6,197 | 11 | 7.0 (5.6, 8.5) | 7.8 (6.2, 9.4) | 6.6 (5.3, 8.2) | 6.7 (6.2, 8.1) | 7.3 (5.9, 8.9) | <0.001 |
|  | Glucose, mg/dL | 3,483 | 50 | 101.0  (87.0, 135.0) | 102.6  (88.2, 138.0) | 99.0  (90.0, 138.6) | 90.0  (86.4, 108.0) | 102.6  (88.2, 136.8) | 0.741 |
|  | HbA1C, mg% | 2,502 | 64 | 6.3 (5.6, 7.3) | 6.3 (5.6, 7.3) | 5.9 (5.4, 6.5) | 6.6 (4.6, 12.4) | 6.3 (5.6, 7.3) | 0.304 |
|  | LDL, mg/dL | 4,179 | 40 | 101.0  (79.0, 128.0) | 104.0  (82.0, 133.0) | 100.5  (83.0, 131.5) | 135.0  (72.0, 217.0) | 102.8  (80.6, 130.0) | 0.042 |
|  | Cholesterol, mg/dL | 6,492 | 7 | 179.0  (150.0, 215.0) | 181.0  (150.0, 217.9) | 157.0  (131.0, 204.0) | 194.0  (175.0, 281.8) | 180.0  (150.0, 216.0) | 0.042 |
|  | Triglyceride, mg/dL | 6,029 | 14 | 115.5  (82.0, 158.0) | 127.4  (92.0, 183.0) | 107.5  (81.0, 159.0) | 207.0  (109.0, 211.6) | 121.2  (86.8, 171.0) | <0.001 |
|  | HDL, mg/dL | 3,990 | 43 | 45.0  (37.0, 56.0) | 45.0  (37.0, 56.0) | 52.0  (47.0, 62.0) | 43.0  (39.0, 53.0) | 45.0  (37.0, 56.0) | 0.114 |
|  | Iron, ug/dL | 2,332 | 67 | 51.0  (34.5, 71.0) | 52.0  (35.2, 72.1) | 54.0  (34.6, 72.0) | 35.0  (35.0, 35.0) | 52.0  (35.0, 72.0) | 0.81 |
|  | TIBC, ug/dL | 2,343 | 66 | 208.9  (172.6, 245.0) | 207.0  (173.0, 246.0) | 230.0  (187.0, 244.0) | 171.3  (171.3, 171.3) | 207.0  (173.0, 246.0) | 0.937 |
|  | Ferritin, ng/dL | 365 | 95 | 188.0  (165.0, 217.0) | 181.0  (156.0, 213.0) | 183.0  (183.0, 203.0) | 268.0  (268.0, 268.0) | 183.0  (157.0, 213.0) | 0.366 |
|  | antiHCV, n(%) | 6,981 | 0 | 38 (1.02) | 41 (1.29) | 0 (0.00) | 0 (0.00) | 79 (1.13) | 0.767 |
|  | antiHBs, n(%) | 6,981 | 0 | 1041 (27.86) | 173 (5.43) | 1 (1.85) | 0 (0.00) | 1215 (17.40) | <0.001 |

**Table S3**. Baseline characteristics of patients included in the PPA by treatments approach (cont.)

| **Outcomes** | **Variables** | **N** | **% Missing** | **Treatment group** | | | | **Total** | **p-value** |
| --- | --- | --- | --- | --- | --- | --- | --- | --- | --- |
|  |  |  |  | **No-PBs** | **CBPBs** | **NCBPBs** | **Aluminum**  **hydroxide** |  |  |
| CVD | **Total number of patients, n (%)** |  |  | 3,233 (59.0) | 2,193 (40.0) | 46 (0.8) | 5 (0.09) | 5,477 (100) |  |
|  | **Hospital, n(%)** | 5,477 | 0 |  |  |  |  |  | <0.001 |
|  | Ramathibodi Hospital |  |  | 2,825 (87.38) | 1,389 (63.34) | 26 (56.52) | 4 (80.00) | 4,244 (77.49) |  |
|  | Bhumibol Hospital |  |  | 408 (12.62) | 804 (36.66) | 20 (43.48) | 1 (20.00) | 1,233 (22.51) |  |
|  | **age (years), mean(SD)** | 5,477 | 0 | 46.23 (16.11) | 59.39 (16.49) | 62.22 (14.35) | 43.43 (10.00) | 51.63 (17.50) | <0.001 |
|  | **Female, n(%)** | 5,477 | 0 | 1,353 (41.85) | 1,187 (54.13) | 20 (43.48) | 2 (40.00) | 2,562 (46.78) | <0.001 |
|  | **Reimbursement, n(%)** | 5,477 | 0 |  |  |  |  |  | <0.001 |
|  | non-CSMBS |  |  | 2,478 (76.65) | 1,319 (60.15) | 11 (23.91) | 4 (80.00) | 3,812 (69.60) |  |
|  | CSMBS |  |  | 755 (23.35) | 874 (39.85) | 35 (76.09) | 1 (20.00) | 1,665 (30.40) |  |
|  | **BMI (kg/m^2^ ), mean(SD)** | 2,391 | 56 | 24.06 (4.68) | 24.72 (5.40) | 26.51 (6.95) | 22.20 (0.57) | 24.25 (4.91) | 0.006 |
|  | **Marital status, n(%)** | 5,477 | 0 |  |  |  |  |  | <0.001 |
|  | Single |  |  | 1,114 (34.46) | 574 (26.17) | 8 (17.39) | 2 (40.00) | 1,698 (31.00) |  |
|  | Married |  |  | 1,833 (56.70) | 1,317 (60.05) | 34 (73.91) | 3 (60.00) | 3,187 (58.19) |  |
|  | Divorced/widowed |  |  | 281 (8.69) | 295 (13.45) | 4 (8.70) | 0 (0.00) | 580 (10.59) |  |
|  | Priest |  |  | 0 (0.00) | 1 (0.05) | 0 (0.00) | 0 (0.00) | 1 (0.02) |  |
|  | Unknown |  |  | 5 (0.15) | 6 (0.27) | 0 (0.00) | 0 (0.00) | 11 (0.20) |  |
|  | **Education levels, n(%)** | 2,887 | 47 |  |  |  |  |  | <0.001 |
|  | Primary level or less |  |  | 503 (21.86) | 166 (29.38) | 6 (30.00) | 0 (0.00) | 675 (23.38) |  |
|  | Secondary level |  |  | 712 (30.94) | 111 (19.65) | 3 (15.00) | 0 (0.00) | 826 (28.61) |  |
|  | University level or more |  |  | 1,086 (47.20) | 288 (50.97) | 11 (55.00) | 1 (100.00) | 1,386 (48.01) |  |
|  | **Renal replacement therapy, n(%)** | 5,477 | 0 |  |  |  |  |  | <0.001 |
|  | No RRT |  |  | 2,007 (62.08) | 1,684 (76.79) | 27 (58.70) | 0 (0.00) | 3,718 (67.88) |  |
|  | RRT |  |  | 1,226 (37.92) | 509 (23.21) | 19 (41.30) | 5 (100.00) | 1,759 (32.12) |  |
|  | **Comorbid, n(%)** |  |  |  |  |  |  |  |  |
|  | Hypertension | 5,477 | 0 | 1,406 (43.49) | 2,091 (95.35) | 36 (78.26) | 5 (100.00) | 3,538 (64.60) | <0.001 |
|  | Diabetes | 5,477 | 0 | 588 (18.19) | 1,024 (46.69) | 18 (39.13) | 1 (20.00) | 1,631 (29.78) | <0.001 |
|  | Dyslipidemia | 5,477 | 0 | 610 (18.87) | 1,358 (61.92) | 25 (54.35) | 2 (40.00) | 1,995 (36.43) | <0.001 |

**Table S3**. Baseline characteristics of patients included in the PPA by treatments approach (cont.)

| **Outcomes** | **Variables** | **N** | **% Missing** | **Treatment group** | | | | **Total** | **p-value** |
| --- | --- | --- | --- | --- | --- | --- | --- | --- | --- |
|  |  |  |  | **No-PBs** | **CBPBs** | **NCBPBs** | **Aluminum**  **hydroxide** |  |  |
| CVD | Bone disorder related ESRD | 5,477 | 0 | 41 (1.27) | 188 (8.57) | 2 (4.35) | 0 (0.00) | 231 (4.22) | <0.001 |
|  | Gout | 5,477 | 0 | 173 (5.35) | 324 (14.77) | 2 (4.35) | 1 (20.00) | 500 (9.13) | <0.001 |
|  | Liver disease | 5,477 | 0 | 108 (3.34) | 103 (4.70) | 3 (6.52) | 1 (20.00) | 215 (3.93) | 0.027 |
|  | SLE | 5,477 | 0 | 38 (1.18) | 59 (2.69) | 1 (2.17) | 0 (0.00) | 98 (1.79) | 0.001 |
|  | Cancer | 5,477 | 0 | 19 (0.59) | 24 (1.09) | 1 (2.17) | 0 (0.00) | 44 (0.80) | 0.072 |
|  | AIDS | 5,477 | 0 | 5 (0.15) | 17 (0.78) | 1 (2.17) | 0 (0.00) | 23 (0.42) | <0.001 |
|  | GI disorder | 5,477 | 0 | 84 (2.60) | 213 (9.71) | 6 (13.04) | 0 (0.00) | 303 (5.53) | <0.001 |
|  | Chronic pulmonary disease | 5,477 | 0 | 23 (0.71) | 41 (1.87) | 1 (2.17) | 0 (0.00) | 65 (1.19) | 0.002 |
|  | Tumor lysis syndrome | 5,477 | 0 | 1 (0.03) | 0 (0.00) | 0 (0.00) | 0 (0.00) | 1 (0.02) | <0.001 |
|  | Hypoparathyroidism | 5,477 | 0 | 0 (0.00) | 2 (0.09) | 0 (0.00) | 0 (0.00) | 2 (0.04) | 0.558 |
|  | Secondary  Hyperparathyroidism | 5,477 | 0 | 220 (6.80) | 636 (29.00) | 26 (56.5) | 3 (60.00) | 885 (16.16) | <0.001 |
|  | **Laboratory, median (IQR)** |  |  |  |  |  |  |  |  |
|  | eGFR, ml/min/1.73 m^2^ | 5,461 | 0 | 4.8 (3.6, 7.2) | 7.4 (4.6, 11.8) | 5.4 (4.2, 8.8) | 3.4 (3.4, 4.0) | 5.4 (3.8, 9.2) | <0.001 |
|  | Phosphorus, mg/dL | 5,477 | 0 | 5.6 (5.0, 6.6) | 5.4 (4.8, 6.4) | 5.7 (5.0, 7.0) | 7.8 (6.6, 8.1) | 5.6 (5.0, 6.6) | <0.001 |
|  | PTH, pg/dL | 3,153 | 42 | 341.8  (157.0, 658.0) | 181.0  (84.0, 358.6) | 400.2  (190.8, 852.8) | 943.5  (44.0, 2783.5) | 271.4  (120.0, 553.0) | <0.001 |
|  | Calcium, mg/dL | 5,477 | 0 | 9.6 (9.1, 10.2) | 9.4 (8.9, 9.9) | 10.2 (9.7, 10.6) | 10.7 (10.6, 11.8) | 9.6 (9.0, 10.1) | <0.001 |
|  | Hemoglobin, g/dL | 4,825 | 12 | 10.6  (9.1, 11.9) | 9.6  (8.3, 11.0) | 10.8  (9.3, 11.7) | 9.8  (9.2, 10.7) | 10.2  (8.8, 11.6) | <0.001 |
|  | Hematocrit, % | 1,432 | 74 | 28.9  (24.6, 33.0) | 28.4  (24.0, 32.7) | 32.2  (29.0, 36.4) | 31.4  (31.4, 31.4) | 28.7  (24.1, 32.9) | 0.799 |
|  | Albumin, g/dL | 5,477 | 0 | 3.7 (3.2, 4.1) | 3.5 (2.9, 3.9) | 3.6 (3.3, 4.1) | 3.3 (3.2, 3.3) | 3.6 (3.1, 4.0) |  |
|  | Sodium, mEq/L | 5,429 | 1 | 138.0  (136.0, 140.0) | 138.0  (135.0, 140.0) | 139.0  (136.0, 142.0) | 139.0  (138.0, 140.0) | 138.0  (135.0, 140.0) | 1.000 |
|  | Potassium, mEq/L | 2,520 | 54 | 4.6 (4.1, 5.2) | 4.6 (4.1, 5.0) | 4.4 (4.0, 5.0) | 3.9 (3.4, 4.4) | 4.6 (4.1, 5.1) | <0.001 |

**Table S3**. Baseline characteristics of patients included in the PPA by treatments approach (cont.)

| **Outcomes** | **Variables** | **N** | **% Missing** | **Treatment group** | | | | **Total** | **p-value** |
| --- | --- | --- | --- | --- | --- | --- | --- | --- | --- |
|  |  |  |  | **No-PBs** | **CBPBs** | **NCBPBs** | **Aluminum**  **hydroxide** |  |  |
| CVD | Chloride, mEq/L | 5,434 | 1 | 100.0  (97.0, 103.0) | 103.0  (99.0, 107.0) | 100.5  (98.0, 105.0) | 99.0  (98.0, 100.0) | 101.0  (98.0, 105.0) | <0.001 |
|  | Carbondioxide, mEq/L | 5,439 | 1 | 23.0  (20.2, 25.8) | 21.0  (18.0, 24.5) | 21.0  (19.0, 24.0) | 22.0  (16.8, 25.7) | 22.3  (19.0, 25.2) | <0.001 |
|  | Uric, mg/dL | 4,843 | 12 | 7.0  (5.6, 8.4) | 7.7  (6.1, 9.3) | 6.6  (5.3, 8.3) | 7.9  (3.3, 9.0) | 7.2  (5.8, 8.8) | <0.001 |
|  | Glucose, mg/dL | 2,494 | 54 | 100.4  (86.4, 131.0) | 102.0  (88.2, 134.0) | 98.0  (90.0, 140.0) | 85.5  (81.0, 90.0) | 101.0  (87.0, 133.2) | 0.779 |
|  | HbA1C, mg% | 1,582 | 71 | 6.3 (5.6, 7.3) | 6.2 (5.5, 7.3) | 5.9 (5.3, 6.5) | 4.6 (4.6, 4.6) | 6.2 (5.5, 7.3) | 0.096 |
|  | LDL, mg/dL | 2,975 | 46 | 104.0  (81.0, 129.0) | 108.0  (85.0, 137.0) | 104.0  (90.8, 148.0) | 72.0  (72.0, 72.0) | 106.0  (83.0, 133.0) | 0.061 |
|  | Cholesterol, mg/dL | 5,024 | 8 | 180.8  (152.6, 216.0) | 185.0  (154.0, 221.0) | 164.0  (138.0, 222.0) | 182.5  (174.5, 235.9) | 182.0  (153.0, 218.0) | 0.105 |
|  | Triglyceride, mg/dL | 4,658 | 15 | 115.0  (82.0, 157.0) | 128.4  (92.0, 183.0) | 116.0  (86.0, 173.0) | 160.3  (109.0, 211.6) | 121.0  (86.0, 169.0) | <0.001 |
|  | HDL, mg/dL | 2,846 | 48 | 46.0  (37.0, 56.0) | 45.1  (38.0, 58.0) | 53.0  (45.0, 70.0) | 39.0  (39.0, 39.0) | 46.0  (37.8, 57.0) | 1.000 |
|  | Iron, ug/dL | 1,555 | 72 | 52.8  (36.0, 74.0) | 54.5  (38.0, 75.0) | 61.0  (34.6, 72.0) | NA | 54.0  (37.4, 75.0) | 0.124 |
|  | TIBC, ug/dL | 1,561 | 71 | 209.0  (174.3, 244.0) | 207.0  (176.0, 246.0) | 234.0  (198.0, 246.0) | NA | 209.0  (176.0, 245.0) | 0.211 |
|  | Ferritin, ng/dL | 284 | 95 | 190.0  (166.0, 218.0) | 181.0  (156.0, 212.0) | 183.0  (183.0, 203.0) | NA | 183.0  (157.0, 212.5) | 0.406 |
|  | antiHCV, n(%) | 5,477 | 0 | 30 (0.93) | 29 (1.32) | 0 (0.00) | 0 (0.00) | 59 (1.08) | 0.650 |
|  | antiHBs, n(%) | 5,477 | 0 | 983 (30.41) | 89 (4.06) | 1 (2.17) | 0 (0.00) | 1073 (19.59) | <0.001 |

**Table S3**. Baseline characteristics of patients included in the PPA by treatments approach (cont.)

| **Outcomes** | **Variables** | **N** | **% Missing** | **Treatment group** | | | | **Total** | **p-value** |
| --- | --- | --- | --- | --- | --- | --- | --- | --- | --- |
|  |  |  |  | **No-PBs** | **CBPBs** | **NCBPBs** | **Aluminum**  **hydroxide** |  |  |
| Bone disorder | **Total number of patients, n (%)** |  |  | **3,658** | **2,821** | **52** | **7** | **6,538** |  |
|  | **Hospital, n(%)** | 6,538 | 0 |  |  |  |  |  | <0.001 |
|  | Ramathibodi Hospital |  |  | 3,229 (88.27) | 1,905 (67.53) | 36 (69.23) | 4 (57.14) | 5,174 (79.14) |  |
|  | Bhumibol Hospital |  |  | 429 (11.73) | 916 (32.47) | 16 (30.77) | 3 (42.86) | 1,364 (20.86) |  |
|  | **age (years), mean(SD)** | 6,538 | 0 | 48.05 (16.83) | 60.74 (15.96) | 62.73 (14.73) | 53.11 (16.60) | 53.65 (17.61) | <0.001 |
|  | **Female, n(%)** | 6,538 | 0 | 1,510 (41.28) | 1,426 (50.55) | 22 (42.31) | 4 (57.14) | 2,962 (45.30) | <0.001 |
|  | **Reimbursement, n(%)** | 6,538 | 0 |  |  |  |  |  | <0.001 |
|  | non-CSMBS |  |  | 2,691 (73.56) | 1,627 (57.67) | 12 (23.08) | 6 (85.71) | 4,336 (66.32) |  |
|  | CSMBS |  |  | 967 (26.44) | 1,194 (42.33) | 40 (76.92) | 1 (14.29) | 2,202 (33.68) |  |
|  | **BMI (kg/m^2^ ), mean(SD)** | 2,840 | 57 | 24.13 (4.73) | 24.97 (5.42) | 23.75 (5.13) | 23.35 (2.19) | 24.38 (4.97) | 0.001 |
|  | **Marital status, n(%)** | 6,538 | 0 |  |  |  |  |  | <0.001 |
|  | Single |  |  | 1,180 (32.26) | 657 (23.29) | 9 (17.31) | 4 (57.14) | 1,850 (28.30) |  |
|  | Married |  |  | 2,128 (58.17) | 1,765 (62.57) | 35 (67.31) | 3 (42.86) | 3,931 (60.13) |  |
|  | Divorced/widowed |  |  | 346 (9.46) | 389 (13.79) | 8 (15.38) | 0 (0.00) | 743 (11.36) |  |
|  | Priest |  |  | 0 (0.00) | 2 (0.07) | 0 (0.00) | 0 (0.00) | 2 (0.03) |  |
|  | Unknown |  |  | 4 (0.11) | 8 (0.28) | 0 (0.00) | 0 (0.00) | 12 (0.18) |  |
|  | **Education levels, n(%)** | 3,283 | 50 |  |  |  |  |  | <0.001 |
|  | Primary level or less |  |  | 580 (23.00) | 230 (31.21) | 5 (21.74) | 0 (0.00) | 815 (24.82) |  |
|  | Secondary level |  |  | 760 (30.13) | 156 (21.17) | 3 (13.04) | 0 (0.00) | 919 (27.99) |  |
|  | University level or more |  |  | 1,182 (46.87) | 351 (47.63) | 15 (65.22) | 1 (100.00) | 1,549 (47.18) |  |
|  | **Renal replacement therapy, n(%)** | 6,538 | 0 |  |  |  |  |  | <0.001 |
|  | No RRT |  |  | 2,268 (62.00) | 2,110 (74.80) | 23 (44.23) | 2 (28.57) | 4,403 (67.34) |  |
|  | RRT |  |  | 1,390 (38.00) | 711 (25.20) | 29 (55.77) | 5 (71.43) | 2,135 (32.66) |  |
|  | **Comorbid, n(%)** |  |  |  |  |  |  |  |  |
|  | Hypertension | 6,538 | 0 | 1,803 (49.29) | 2,720 (96.42) | 42 (80.77) | 7 (100.00) | 4,572 (69.93) | <0.001 |
|  | Diabetes | 6,538 | 0 | 837 (22.88) | 1,485 (52.64) | 19 (36.54) | 3 (42.86) | 2,344 (35.85) | <0.001 |
|  | Dyslipidemia | 6,538 | 0 | 911 (24.90) | 1,937 (68.66) | 31 (59.62) | 4 (57.14) | 2,883 (44.10) | <0.001 |

**Table S3**. Baseline characteristics of patients included in the PPA by treatments approach (cont.)

| **Outcomes** | **Variables** | **N** | **%**  **Missing** | **Treatment group** | | | | **Total** | **p-value** |
| --- | --- | --- | --- | --- | --- | --- | --- | --- | --- |
|  |  |  |  | **No-PBs** | **CBPBs** | **NCBPBs** | **Aluminum**  **hydroxide** |  |  |
| Bone disorder | Bone disorder related ESRD | 6,538 | 0 | 477 (13.04) | 862 (30.56) | 13 (25.00) | 3 (42.86) | 1,355 (20.72) | <0.001 |
|  | Gout | 6,538 | 0 | 128 (3.50) | 200 (7.09) | 1 (1.92) | 0 (0.00) | 329 (5.03) | <0.001 |
|  | Liver disease | 6,538 | 0 | 143 (3.91) | 161 (5.71) | 3 (5.77) | 0 (0.00) | 307 (4.70) | 0.015 |
|  | SLE | 6,538 | 0 | 49 (1.34) | 61 (2.16) | 1 (1.92) | 0 (0.00) | 111 (1.70) | 0.101 |
|  | Cancer | 6,538 | 0 | 25 (0.68) | 26 (0.92) | 0 (0.00) | 0 (0.00) | 51 (0.78) | 0.802 |
|  | AIDS | 6,538 | 0 | 6 (0.16) | 21 (0.74) | 1 (1.92) | 0 (0.00) | 28 (0.43) | 0.001 |
|  | GI disorder | 6,538 | 0 | 115 (3.14) | 294 (10.42) | 4 (7.69) | 2 (28.57) | 415 (6.35) | <0.001 |
|  | Chronic pulmonary disease | 6,538 | 0 | 47 (1.28) | 90 (3.19) | 2 (3.85) | 0 (0.00) | 139 (2.13) | <0.001 |
|  | Tumor lysis syndrome | 6,538 | 0 | 2 (0.05) | 0 (0.00) | 0 (0.00) | 0 (0.00) | 2 (0.03) | 0.813 |
|  | Hypoparathyroidism | 6,538 | 0 | 0 (0.00) | 6 (0.21) | 0 (0.00) | 0 (0.00) | 6 (0.09) | 0.095 |
|  | Secondary  hyperparathyroidism | 6,538 | 0 | 275 (7.52) | 788 (27.93) | 30 (57.69) | 2 (28.57) | 1,095 (16.75) | <0.001 |
|  | **Laboratory, median (IQR)** |  |  |  |  |  |  |  |  |
|  | eGFR, ml/min/1.73 m^2^ | 6,523 | 0 | 5.0 (3.8, 7.8) | 7.6 (4.8, 11.6) | 5.9 (4.2, 9.8) | 4.6 (3.4, 11.4) | 5.8 (4.0, 10.0) | <0.001 |
|  | Phosphorus, mg/dL | 6,538 | 0 | 5.6 (5.0, 6.6) | 5.3 (4.8, 6.3) | 5.6 (5.0, 6.9) | 6.2 (5.0, 8.1) | 5.4 (4.9, 6.6) | <0.001 |
|  | PTH, pg/dL | 3,583 | 45 | 322.6  (141.8, 640.6) | 174.0  (86.0, 348.8) | 328.5  (140.8, 868.1) | 406.9  (44.0, 1304.4) | 249.6  (112.6, 518.8) | 0.715 |
|  | Calcium, mg/dL | 6,538 | 0 | 9.4 (8.8, 10.0) | 9.3 (8.7, 9.8) | 10.0 (9.6, 10.5) | 10.4 (9.9, 11.8) | 9.4 (8.8, 9.9) | <0.001 |
|  | Hemoglobin, g/dL | 6,538 | 0 | 10.6  (9.1, 11.9) | 9.7  (8.4, 11.1) | 10.8  (9.3, 11.7) | 9.6  (8.7, 11.5) | 10.2  (8.8, 11.6) | <0.001 |
|  | Hematocrit, % | 1,733 | 73 | 29.0  (25.0, 33.0) | 28.8  (24.6, 33.0) | 30.3  (28.1, 36.4) | 31.4  (17.3, 38.0) | 29.0  (24.8, 33.0) | <0.001 |
|  | Albumin, g/dL | 6,538 | 0 | 3.6 (3.2, 4.0) | 3.4 (2.9, 3.8) | 3.6 (3.3, 3.9) | 3.3 (3.2, 3.4) | 3.5 (3.0, 3.9) | 0.001 |
|  | Sodium, mEq/L | 6,492 | 1 | 138.0  (136.0, 140.0) | 138.0  (135.0, 140.0) | 139.0  (136.0, 141.0) | 138.0  (136.0, 140.0) | 138.0  (135.0, 140.0) | 0.954 |
|  | Potassium, mEq/L | 3,132 | 52 | 4.6 (4.0, 5.0) | 4.5 (4.1, 5.0) | 4.4 (4.0, 5.0) | 4.4 (3.8, 4.8) | 4.6 (4.1, 5.0) | 0.239 |

**Table S3**. Baseline characteristics of patients included in the PPA by treatments approach (cont.)

| **Outcomes** | **Variables** | **N** | **% Missing** | **Treatment group** | | | | | **p-value** |
| --- | --- | --- | --- | --- | --- | --- | --- | --- | --- |
|  |  |  |  | **No-PBs** | **CBPBs** | **NCBPBs** | **Aluminum**  **hydroxide** | **Total** |  |
| Bone disorders | Chloride, mEq/L | 6,498 | 1 | 100.0  (97.0, 103.0) | 103.0  (99.0, 107.0) | 100.0  (97.0, 104.0) | 100.0  (98.0, 105.0) | 101.0  (98.0, 105.0) | <0.001 |
|  | Carbondioxide, mEq/L | 6,503 | 1 | 22.9  (20.0, 25.6) | 21.0  (18.0, 24.5) | 21.8  (19.1, 24.6) | 22.0  (16.0, 27.5) | 22.0  (19.0, 25.0) | <0.001 |
|  | Uric, mg/dL | 5,785 | 12 | 7.0 (5.6, 8.5) | 7.8 (6.3, 9.4) | 6.6 (5.3, 8.2) | 6.7 (6.2, 8.1) | 7.3 (5.9, 8.9) | <0.001 |
|  | Glucose, mg/dL | 3,115 | 52 | 101.0  (87.0, 135.0) | 102.6  (88.2, 137.0) | 99.0  (90.0, 138.6) | 90.0  (86.4, 108.0) | 102.6  (88.0, 136.8) | <0.001 |
|  | HbA1C, mg% | 2,203 | 66 | 6.3 (5.6, 7.3) | 6.3 (5.6, 7.3) | 6.0 (5.4, 6.7) | 6.6 (4.6, 12.4) | 6.3 (5.6, 7.3) | <0.001 |
|  | LDL, mg/dL | 3,810 | 42 | 101.8  (79.0, 128.0) | 105.0  (82.0, 133.6) | 100.5  (90.8, 139.0) | 135.0  (72.0, 217.0) | 103.0  (81.0, 131.0) | <0.001 |
|  | Cholesterol, mg/dL | 6,059 | 7 | 179.0  (151.0, 215.0) | 181.9  (150.1, 218.0) | 163.0  (125.0, 205.8) | 194.0  (175.0, 281.8) | 180.0  (150.0, 216.0) | 0.001 |
|  | Triglyceride, mg/dL | 5,613 | 14 | 116.0  (82.0, 158.0) | 128.0  (92.0, 184.0) | 107.5  (80.0, 169.0) | 207.0  (109.0, 211.6) | 121.0  (86.0, 170.8) | <0.001 |
|  | HDL, mg/dL | 3,626 | 45 | 45.0  (37.0, 56.0) | 45.0  (37.0, 56.0) | 52.0  (46.0, 62.0) | 43.0  (39.0, 53.0) | 45.0  (37.0, 56.0) | 0.541 |
|  | Iron, ug/dL | 2,073 | 68 | 51.0  (35.0, 71.5) | 52.0  (35.9, 73.0) | 51.0  (34.3, 71.8) | 35.0  (35.0, 35.0) | 52.0  (35.0, 72.0) | 0.003 |
|  | TIBC, ug/dL | 2,083 | 68 | 208.9  (173.0, 245.0) | 207.0  (174.0, 245.0) | 229.0  (179.5, 242.5) | 171.3  (171.3, 171.3) | 207.8  (174.0, 245.0) | 0.250 |
|  | Ferritin, ng/dL | 335 | 95 | 188.0  (165.0, 217.0) | 181.0  (156.0, 212.0) | 183.0  (175.0, 193.0) | 268.0  (268.0, 268.0) | 183.0  (157.0, 212.0) | 0.308 |
|  | antiHCV, n(%) | 6,538 | 0 | 37 (1.01) | 35 (1.24) | 0 (0.00) | 0 (0.00) | 72 (1.10) | 0.077 |
|  | antiHBs, n(%) | 6,538 | 0 | 1038 (28.38) | 138 (4.89) | 1 (1.92) | 0 (0.00) | 1177 (18.00) | <0.001 |

Abbreviations: Body mass index; (BMI), CBPBs; Calcium-based phosphate binders, CVD; cardiovascular disease, CSMBS; Civil Servant Medical Benefit Scheme, eGFR; estimated Glomerular Filtration Rate, ESRD; End-stage renal disease, HBA1C; Hemoglobin A1C, HDL; High-Density Lipoprotein, HIV; human immunodeficiency virus, IQR; interquartile range, ITT; Intention to treat, GI; Gastrointestinal tract, LDL; Low-Density Lipoprotein, NCBPBs; N; number, Non-calcium-based phosphate binders i.e., lanthanum or sevelamer; OS; overall survival outcome, PBs; Phosphate binders, PPA; Per-protocol analysis, SLE; PTH; Parathyroid hormone levels, RRT, Renal Replacement Therapy, Systemic lupus erythematosus, TIBC; Total iron binding capacity

# Table S4. Baseline characteristics of patients included in the actual treatment-pattern analysis by treatment approach

| **Outcomes** | **Variables** | **N** | **% Missing** | **Treatment group** | | | | **Total** | **p-value** |
| --- | --- | --- | --- | --- | --- | --- | --- | --- | --- |
|  |  |  |  | **No-PBs** | **CBPBs-CBPBs** | **CBPBs-NCBPBs** | **CBPBs-aluminum** |  |  |
| OS | **Total number of patients, n (%)** | 8,005 | 0 | 3,736 (48.6) | 3,361 (43.8) | 487 (6.34) | 98 (1.28) | 7,682 |  |
|  | **Hospital, n(%)** | 7,682 | 0 |  |  |  |  |  | <0.001 |
|  | Ramathibodi Hospital |  |  | 3,283 (87.9) | 2,315 (68.9) | 326 (66.94) | 37 (37.8) | 5,961 (77.6) |  |
|  | Bhumibol Hospital |  |  | 453 (12.1) | 1,046 (31.1) | 161 (33.06) | 61 (62.2) | 1,721 (22.4) |  |
|  | **age (years), mean(SD)** | 7,682 | 0 | 48.5 (17.1) | 61.8 (16.1) | 60.84 (14.74) | 53.6 (15.4) | 55.4 (17.8) | <0.001 |
|  | **Male, n(%)** | 7,682 | 0 | 2,172(58.1) | 1,575 (46.9) | 251 (51.5) | 49 (50.0) | 4,047 (52.7) | <0.001 |
|  | **Reimbursement, n(%)** | 7,682 | 0 |  |  |  |  |  | <0.001 |
|  | non-CSMBS |  |  | 2,723 (72.9) | 1,835 (54.6) | 97 (19.92) | 84 (85.7) | 4,739 (61.7) |  |
|  | CSMBS |  |  | 1,013 (27.1) | 1,526 (45.4) | 390 (80.08) | 14 (14.3) | 2,943 (38.3) |  |
|  | **BMI (kg/m^2^ ), mean(SD)** | 3,288 | 57 | 24.2 (4.7) | 24.9 (5.3) | 25.35 (4.80) | 23.2 (4.2) | 24.47 (4.9) | <0.001 |
|  | **Marital status, n(%)** | 7,682 | 0 |  |  |  |  |  | <0.001 |
|  | Single |  |  | 1,190 (31.9) | 748 (22.3) | 80 (16.43) | 37 (37.8) | 2,055 (26.8) |  |
|  | Married |  |  | 2,178 (58.3) | 2,121 (63.1) | 345 (70.84) | 48 (49.0) | 4,692 (61.1) |  |
|  | Divorced/widowed |  |  | 364 (9.7) | 481 (14.3) | 61 (12.53) | 13 (13.3) | 919 (12.0) |  |
|  | Priest |  |  | 0 (0.0) | 2 (0.1) | 0 (0.00) | 0 (0.00) | 2 (0.03) |  |
|  | Unknown |  |  | 4 (0.1) | 9 (0.3) | 1 (0.21) | 0 (0.00) | 14 (0.2) |  |
|  | **Education levels, n(%)** | 3,508 | 54 |  |  |  |  |  | <0.001 |
|  | Primary level or less |  |  | 589 (23.2) | 270 (32.1) | 29 (25.00) | 4 (57.1) | 892 (25.4) |  |
|  | Secondary level |  |  | 766 (30.1) | 180 (21.4) | 10 (8.62) | 1 (14.3) | 957 (27.3) |  |
|  | University level or more |  |  | 1,189 (46.7) | 391 (46.5) | 77 (66.38) | 2 (28.6) | 1,659 (47.3) |  |
|  | **Renal replacement therapy, n(%)** | 7,682 | 0 |  |  |  |  |  | <0.001 |
|  | No RRT |  |  | 2,333 (62.5) | 2,526 (75.2) | 359 (73.72) | 33 (33.7) | 5,061 (65.9) |  |
|  | RRT |  |  | 1,403 (37.6) | 835 (24.8) | 128 (26.28) | 65 (66.3) | 2,621 (34.1) |  |
|  | **Comorbid, n(%)** |  |  |  |  |  |  |  |  |
|  | Hypertension | 7,682 | 0 | 1,874 (50.2) | 3,247 (96.6) | 480 (98.56) | 92 (93.9) | 5,693 (74.1) | <0.001 |
|  | Diabetes | 7,682 | 0 | 871 (23.3) | 1,806 (53.7) | 253 (51.95) | 38 (38.8) | 2,968 (38.6) | <0.001 |
|  | Dyslipidemia | 7,682 | 0 | 965 (25.8) | 2,351 (70.0) | 360 (73.92) | 48 (49.0) | 3,724 (48.5) | <0.001 |

**Table S4** Baseline characteristics of patients included in the actual treatment-pattern analysis by treatment approach (cont.)

| **Outcomes** | **Variables** | **N** | **% Missing** | **Treatment group** | | | | **Total** | **p-value** |
| --- | --- | --- | --- | --- | --- | --- | --- | --- | --- |
|  |  |  |  | **No-PBs** | **CBPBs-CBPBs** | **CBPBs-NCBPBs** | **CBPBs-aluminum** |  |  |
| OS | CVD | 7,682 | 0 | 503 (13.5) | 912 (27.1) | 132 (27.10) | 13 (13.3) | 1,675 (21.8) | <0.001 |
|  | Bone disorder related ESRD | 7,682 | 0 | 78 (2.1) | 343 (10.2) | 49 (10.06) | 7 (7.1) | 515 (6.7) | <0.001 |
|  | Gout | 7,682 | 0 | 141 (3.8) | 250 (7.4) | 37 (7.60) | 3 (3.1) | 431 (5.6) | <0.001 |
|  | Liver disease | 7,682 | 0 | 150 (4.0) | 216 (6.4) | 32 (6.57) | 2 (2.0) | 400 (5.2) | <0.001 |
|  | SLE | 7,682 | 0 | 50 (1.3) | 81 (2.4) | 6 (1.23) | 1 (1.0) | 138 (1.8) | 0.01 |
|  | Cancer | 7,682 | 0 | 27 (0.7) | 38 (1.1) | 1 (0.21) | 0 (0.0) | 66 (0.9) | 0.122 |
|  | AIDS | 7,682 | 0 | 8 (0.2) | 24 (0.7) | 1 (0.21) | 0 (0.0) | 33 (0.4) | 0.018 |
|  | GI disorder | 7,682 | 0 | 127 (3.4) | 395 (11.8) | 54 (11.09) | 9 (9.2) | 585 (7.6) | <0.001 |
|  | Chronic pulmonary disease | 7,682 | 0 | 55 (1.5) | 131 (3.9) | 18 (3.70) | 2 (2.0) | 206 (2.7) | <0.001 |
|  | Tumor lysis syndrome | 7,682 | 0 | 2 (0.1) | 0 (0.0) | 0 (0.00) | 0 (0.0) | 2 (0.03) | <0.001 |
|  | Hypoparathyroidism | 7,682 | 0 | 0 (0.0) | 8 (0.2) | 0 (0.00) | 0 (0.0) | 8 (0.1) | 0.715 |
|  | Secondary  hyperparathyroidism | 7,682 | 0 | 314 (8.4) | 979 (29.1) | 150 (30.80) | 23 (23.5) | 1,466 (19.1) | <0.001 |
|  | **Laboratory, median (IQR)** |  |  |  |  |  |  |  |  |
|  | eGFR, ml/min/1.73 m^2^ | 7,667 | 0 | 5.0 (3.8, 8.0) | 7.8 (4.8, 12.0) | 6.4 (4.4, 9.8) | 4.6 (3.4, 6.8) | 6.0 (4.0, 10.2) | <0.001 |
|  | Phosphorus, mg/dL | 7,682 | 0 | 5.6 (5.0, 6.6) | 5.5 (5.0, 6.3) | 5.91 (1.61) | 6.6 (6, 7.6) | 5.7 (5.0, 6.6) | <0.001 |
|  | PTH, pg/dL | 4,364 | 43 | 319.6  (141.8, 639.2) | 168.4  (81.0, 335.0) | 319.59  (165.4, 696.1) | 314  (161.6, 705.3) | 242.7  (110.2, 509.3) | <0.001 |
|  | Calcium, mg/dL | 7,682 | 0 | 9.4 (8.8, 10.0) | 9.4 (8.8, 9.9) | 9.33 (1.03) | 9.9 (9.4, 10.6) | 9.4 (8.8, 10.0) | 0.002 |
|  | Hemoglobin, g/dL | 7,682 | 0 | 10.5 (9.1, 11.9) | 9.7 (8.4, 11.1) | 10.3 (8.9, 11.4) | 9.2 (7.4, 10.5) | 10.1 (8.8, 11.5) | <0.001 |
|  | Hematocrit, % | 2,204 | 71 | 29.0  (25.0, 33.0) | 29.0  (24.8, 33.0) | 31.2  (26.4, 34.6) | 28.2  (23.5, 31.4) | 29.1  (25.0, 33.1) | 0.001 |
|  | Albumin, g/dL | 7,682 | 0 | 3.6 (3.1, 4.0) | 3.4 (2.9, 3.8) | 3.6 (3.1, 4.0) | 4.1 (3.7, 4.5) | 3.5 (3.0, 3.9) | <0.001 |
|  | Sodium, mEq/L | 7,639 | 1 | 138.0  (136.0, 140.0) | 138.0  (134.0, 140.0) | 138.0  (135.0, 141.0) | 137.5  (135.0, 140.0) | 138.0  (135.0, 140.0) | <0.001 |
|  | Potassium, mEq/L | 4,025 | 48 | 4.6 (4.0, 5.0) | 4.5 (4.0, 5.0) | 4.6 (4.2, 5.0) | 4.7 (4.2, 5.2) | 4.6 (4.0, 5.0) | 0.004 |
|  | Chloride, mEq/L | 7,645 | 0 | 100.0  (97.0, 104.0) | 103.0  (99.0, 107.0) | 103.0  (99.0, 106.0) | 100.0  (97.0, 105.0) | 101.0  (98.0, 105.0) | <0.001 |

**Table S4** Baseline characteristics of patients included in the actual treatment-pattern analysis by treatment approach (cont.)

| **Outcomes** | **Variables** | **N** | **% Missing** | **Treatment group** | | | | **Total** | **p-value** |
| --- | --- | --- | --- | --- | --- | --- | --- | --- | --- |
|  |  |  |  | **No-PBs** | **CBPBs-CBPBs** | **CBPBs-NCBPBs** | **CBPBs-aluminum** |  |  |
| OS | Carbondioxide, mEq/L | 7,650 | 0 | 22.8  (20.0, 25.5) | 21.0  (18.0, 24.4) | 21.4  (18.6, 24.5) | 21.0  (18.0, 24.0) | 22.0  (19.0, 25.0) | <0.001 |
|  | Uric, mg/dL | 6,848 | 11 | 7.0 (5.6, 8.5) | 7.8 (6.2, 9.5) | N=487 | 7.6 (6.2, 9.1) | 7.4 (5.9, 9.0) | <0.001 |
|  | Glucose, mg/dL | 4,037 | 47 | 101.0  (87.0, 135.0) | 102.6  (88.2, 137.0) | 102.0  (90.0, 136.0) | 101.0  (89.0, 128.0) | 102.6  (88.2, 136.8) | 0.769 |
|  | HbA1C, mg% | 2,851 | 63 | 6.3 (5.6, 7.3) | 6.3 (5.6, 7.3) | 6.2 (5.4, 6.9) | 6.1 (5.6, 7.4) | 6.3 (5.6, 7.3) | 0.039 |
|  | LDL, mg/dL | 4,682 | 39 | 101.0  (79.0, 128.0) | 104.0  (82.0, 132.8) | 101.0  (78.0, 129.5) | 103.0  (82.0, 135.0) | 102.6  (80.0, 130.0) | 0.043 |
|  | Cholesterol, mg/dL | 7,160 | 7 | 179.0  (150.0, 215.0) | 181.0  (150.0, 218.0) | 177.0  (149.0, 211.0) | 185.0  (152.6, 218.2) | 180.0  (150.0, 216.0) | 0.156 |
|  | Triglyceride, mg/dL | 6,643 | 14 | 115.5  (82.0, 158.0) | 128.0  (92.0, 184.0) | 126.0  (91.0, 178.0) | 127.2  (94.0, 178.0) | 122.0  (87.0, 172.0) | <0.001 |
|  | HDL, mg/dL | 4,479 | 42 | 45.0  (37, 56.0) | 45.0  (37, 56.0) | 45.0  (37.0, 55.0) | 49.0  (42.0, 55.0) | 45.0  (37.0, 56.0) | 0.868 |
|  | Iron, ug/dL | 2,746 | 64 | 51.0  (34.5, 71.0) | 52.0  (35.0, 73.0) | 56.5  (41.9, 78.0) | 56.0  (42.5, 75.0) | 52.0  (36.0, 73.0) | 0.153 |
|  | TIBC, ug/dL | 2,759 | 64 | 208.9  (172.6, 245.0) | 206.0  (173.0, 246.9) | 210.8  (171.3, 243.8) | 214.0  (185.0, 255.0) | 207.0  (173.0, 246.0) | 0.793 |
|  | Ferritin, ng/dL | 441 | 94 | 188.0  (165.0, 217.0) | 181.5  (156.0, 213.0) | 175.0  (159.0, 196.0) | 193.0  (158.0, 211.5) | 182.0  (157.0, 211.0) | 0.775 |
|  | antiHCV | 7,682 | 0 | 38 (1.02) | 44 (1.31) | 4 (0.82) | 3 (3.06) | 89 (1.16) | 0.297 |
|  | antiHBs | 7,682 | 0 | 1041 (27.86) | 184 (5.47) | 25 (5.13) | 2 (2.04) | 1252 (16.30) | <0.001 |

**Table S4** Baseline characteristics of patients included in the actual treatment-pattern analysis by treatment approach (cont.)

| **Outcomes** | **Variables** | **N** | **% Missing** | **Treatment group** | | | | **Total** | **p-value** |
| --- | --- | --- | --- | --- | --- | --- | --- | --- | --- |
|  |  |  |  | **No-PBs** | **CBPBs-CBPBs** | **CBPBs-NCBPBs** | **CBPBs-aluminum** |  |  |
| **CVD** | **Total number of patients, n (%)** |  |  | 3,233 (53.8) | 2,449 (40.8) | 240 (4.0) | 85 (1.4) | 6,007 (100.0) |  |
|  | **Hospital, n(%)** | 6,007 | 0 |  |  |  |  |  | <0.001 |
|  | Ramathibodi Hospital |  |  | 2,825 (87.38) | 1,575 (64.31) | 130 (54.17) | 25 (29.41) | 4,555 (75.83) |  |
|  | Bhumibol Hospital |  |  | 408 (12.62) | 874 (35.69) | 110 (45.83) | 60 (70.59) | 1,452 (24.17) |  |
|  | **age (years), mean(SD)** | 6,007 | 0 | 46.23 (16.11) | 59.45 (16.21) | 56.88 (14.48) | 50.69 (15.47) | 52.20 (17.36) | <0.001 |
|  | **Female, n(%)** | 6,007 | 0 | 1,353 (41.85) | 1,314 (53.65) | 118 (49.17) | 42 (49.41) | 2,827 (47.06) | <0.001 |
|  | **Reimbursement, n(%)** | 6,007 | 0 |  |  |  |  |  | <0.001 |
|  | non-CSMBS |  |  | 2,478 (76.65) | 1,405 (57.37) | 55 (22.92) | 77 (90.59) | 4,002 (66.62) |  |
|  | CSMBS |  |  | 755 (23.35) | 1,044 (42.63) | 185 (77.08) | 8 (9.41) | 2,005 (33.38) |  |
|  | **BMI (kg/m^2^ ), mean(SD)** | 2,602 | 57 | 24.06 (4.68) | 24.70 (5.30) | 25.30 (4.99) | 23.97 (4.49) | 24.27 (4.86) | 0.029 |
|  | **Marital status, n(%)** | 6,007 | 0 |  |  |  |  |  | <0.001 |
|  | Single |  |  | 1,114 (34.46) | 614 (25.07) | 46 (19.17) | 41 (48.24) | 1,815 (30.21) |  |
|  | Married |  |  | 1,833 (56.70) | 1,495 (61.05) | 163 (67.92) | 32 (37.65) | 3,523 (58.65) |  |
|  | Divorced/widowed |  |  | 281 (8.69) | 333 (13.60) | 30 (12.50) | 12 (14.12) | 656 (10.92) |  |
|  | Priest |  |  | 0 (0.00) | 1 (0.04) | 0 (0.00) | 0 (0.00) | 1 (0.02) |  |
|  | Unknown |  |  | 5 (0.15) | 6 (0.24) | 1 (0.42) | 0 (0.00) | 12 (0.20) |  |
|  | **Education levels, n(%)** | 2,988 | 50 |  |  |  |  |  | <0.001 |
|  | Primary level or less |  |  | 503 (21.86) | 186 (29.48) | 12 (25.53) | 2 (22.22) | 703 (23.53) |  |
|  | Secondary level |  |  | 712 (30.94) | 123 (19.49) | 5 (10.64) | 3 (33.33) | 843 (28.21) |  |
|  | University level or more |  |  | 1,086 (47.20) | 322 (51.03) | 30 (63.83) | 4 (44.44) | 1,442 (48.26) |  |
|  | **Renal replacement therapy, n(%)** | 6,007 | 0 |  |  |  |  |  | <0.001 |
|  | No RRT |  |  | 2,007 (62.08) | 1,874 (76.52) | 187 (77.92) | 41 (48.24) | 4,023 (66.97) |  |
|  | RRT |  |  | 1,226 (37.92) | 575 (23.48) | 53 (22.08) | 44 (51.76) | 1,984 (33.03) |  |
|  | **Comorbid, n(%)** |  |  |  |  |  |  |  |  |
|  | Hypertension | 6,007 | 0 | 1,406 (43.49) | 2,341 (95.59) | 237 (98.75) | 84 (98.82) | 4,071 (67.77) | <0.001 |
|  | Diabetes | 6,007 | 0 | 588 (18.19) | 1,144 (46.71) | 103 (42.92) | 38 (44.71) | 1,890 (31.46) | <0.001 |

**Table S4** Baseline characteristics of patients included in the actual treatment-pattern analysis by treatment approach (cont.)

| **Outcomes** | **Variables** | **N** | **% Missing** | **Treatment group** | | | | **Total** | **p-value** |
| --- | --- | --- | --- | --- | --- | --- | --- | --- | --- |
|  |  |  |  | **No-PBs** | **CBPBs-CBPBs** | **CBPBs-NCBPBs** | **CBPBs-aluminum** |  |  |
| CVD | Diabetes | 6,007 | 0 | 588 (18.19) | 1,144 (46.71) | 103 (42.92) | 38 (44.71) | 1,890 (31.46) | <0.001 |
|  | Dyslipidemia | 6,007 | 0 | 610 (18.87) | 1,526 (62.31) | 158 (65.83) | 40 (47.06) | 2,357 (39.24) | <0.001 |
|  | Bone disorder related ESRD | 6,007 | 0 | 41 (1.27) | 214 (8.74) | 16 (6.67) | 2 (2.35) | 285 (4.74) | <0.001 |
|  | Gout | 6,007 | 0 | 173 (5.35) | 366 (14.94) | 15 (6.25) | 13 (15.29) | 611 (10.17) | <0.001 |
|  | Liver disease | 6,007 | 0 | 108 (3.34) | 115 (4.70) | 19 (7.92) | 5 (5.88) | 254 (4.23) | <0.001 |
|  | SLE | 6,007 | 0 | 38 (1.18) | 64 (2.61) | 2 (0.83) | 5 (5.88) | 113 (1.88) | <0.001 |
|  | Cancer | 6,007 | 0 | 19 (0.59) | 24 (0.98) | 0 (0.00) | 0 (0.00) | 43 (0.72) | 0.240 |
|  | AIDS | 6,007 | 0 | 5 (0.15) | 19 (0.78) | 1 (0.42) | 1 (1.18) | 27 (0.45) | 0.008 |
|  | GI disorder | 6,007 | 0 | 84 (2.60) | 238 (9.72) | 26 (10.83) | 14 (16.47) | 377 (6.28) | <0.001 |
|  | Chronic pulmonary disease | 6,007 | 0 | 23 (0.71) | 44 (1.80) | 8 (3.33) | 0 (0.00) | 78 (1.30) | <0.001 |
|  | Tumor lysis syndrome | 6,007 | 0 | 1 (0.03) | 0 (0.00) | 0 (0.00) | 0 (0.00) | 1 (0.02) | 0.930 |
|  | Hypoparathyroidism | 6,007 | 0 | 0 (0.00) | 3 (0.12) | 0 (0.00) | 1 (1.18) | 7 (0.12) | <0.001 |
|  | Secondary  Hyperparathyroidism | 6,007 | 0 | 220 (6.80) | 719 (29.36) | 73 (30.42) | 53 (62.35) | 1,153 (19.19) | <0.001 |
|  | **Laboratory, median(IQR)** |  |  |  |  |  |  |  |  |
|  | eGFR, ml/min/1.73 m^2^ | 5,997 | 0 | 4.8 (3.6, 7.2) | 7.2 (4.6, 11.6) | 5.8 (4.2, 9.4) | 4.0 (3.2, 5.4) | 5.4 (3.8, 9.0) | <0.001 |
|  | Phosphorus, mg/dL | 6,007 | 0 | 5.6 (5.0, 6.6) | 5.4 (4.8, 6.4) | 5.4 (4.9, 6.6) | 6.6 (5.7, 7.5) | 5.6 (5.0, 6.6) | <0.001 |
|  | PTH, pg/dL | 3,575 | 40 | 341.8  (157.0, 658.0) | 183.0  (86.4, 365.0) | 257.6  (118.0, 519.8) | 350.0  (170.0, 701.6) | 269.6  (120.0, 544.0) | <0.001 |
|  | Calcium, mg/dL | 6,007 | 0 | 9.6  (9.1, 10.2) | 9.4  (8.9, 9.9) | 9.4  (9.0, 10.0) | 10.3  (9.6, 10.8) | 9.6  (9.0, 10.1) | <0.001 |
|  | Hemoglobin, g/dL | 5,296 | 12 | 10.6 (9.1, 11.9) | 9.7 (8.4, 11.0) | 10.2 (8.7, 11.2) | 10.2 (8.8, 11.3) | 10.2 (8.8, 11.6) | <0.001 |
|  | Hematocrit, % | 1,693 | 72 | 28.9  (24.6, 33.0) | 28.5  (24.0, 32.7) | 31.2  (26.4, 34.4) | 31.5  (27.9, 34.2) | 29.1  (24.6, 33.1) | <0.001 |
|  | Albumin, g/dL | 6,007 | 0 | 3.7 (3.2, 4.1) | 3.5 (2.9, 3.9) | 3.7 (3.3, 4.2) | 4.2 (3.6, 4.6) | 3.6 (3.1, 4.0) | 0.815 |

**Table S4** Baseline characteristics of patients included in the actual treatment-pattern analysis by treatment approach (cont.)

| **Outcomes** | **Variables** | **N** | **% Missing** | **Treatment group** | | | | **Total** | **p-value** |
| --- | --- | --- | --- | --- | --- | --- | --- | --- | --- |
|  |  |  |  | **No-PBs** | **CBPBs-CBPBs** | **CBPBs-NCBPBs** | **CBPBs-aluminum** |  |  |
| CVD | Chloride, mEq/L | 5,973 | 1 | 100.0  (97.0, 103.0) | 103.0  (99.0, 107.0) | 103.0  (99.0, 106.0) | 98.0  (95.0, 101.0) | 101.0  (97.0, 105.0) | <0.001 |
|  | Uric, mg/dL | 5,363 | 11% | 7.0 (5.6, 8.4) | 7.8 (6.2, 9.3) | 7.8 (6.5, 9.6) | 6.9 (5.7, 8.6) | 7.3 (5.9, 8.8) | <0.001 |
|  | Glucose, mg/dL | 2,921 | 51% | 100.4  (86.4, 131.0) | 102.0  (88.2, 134.0) | 99.0  (88.0, 136.0) | 96.0  (86.0, 119.0) | 100.8  (87.0, 133.0) | 0.122 |
|  | HbA1C, mg% | 1,889 | 69% | 6.3 (5.6, 7.3) | 6.2 (5.5, 7.3) | 6.1 (5.4, 6.9) | 5.8 (5.2, 6.7) | 6.2 (5.5, 7.2) | <0.001 |
|  | LDL, mg/dL | 3,395 | 43% | 104.0  (81.0, 129.0) | 108.0  (85.0, 136.2) | 101.5  (80.0, 135.0) | 96.0  (78.0, 134.0) | 105.0  (82.0, 132.8) | 0.296 |
|  | Cholesterol, mg/dL | 5,555 | 8% | 180.8  (152.6, 216.0) | 184.0  (154.0, 221.0) | 179.0  (150.5, 211.0) | 180.0  (149.0, 218.0) | 182.0  (153.0, 218.0) | 1.000 |
|  | Triglyceride, mg/dL | 5,156 | 14% | 115.0  (82.0, 157.0) | 128.0  (92.0, 181.4) | 133.0  (96.4, 187.0) | 117.0  (92.0, 157.0) | 121.0  (86.0, 168.6) | 0.015 |
|  | HDL, mg/dL | 3,254 | 46% | 46.0  (37.0, 56.0) | 45.0  (38, 58.0) | 47.0  (37.0, 57.0) | 49.9  (41.0, 61.0) | 46.0  (37.8, 57.0) | 1.000 |
|  | Iron, ug/dL | 1,941 | 68% | 52.8  (36.0, 74.0) | 54.1  (38.0, 75.0) | 60.0  (44.0, 83.0) | 61.0  (45.0, 82.0) | 55.0  (39.0, 75.0) | 0.008 |
|  | TIBC, ug/dL | 1,950 | 68% | 209.0  (174.3, 244.0) | 207.0  (175.0, 247.5) | 212.5  (172.2, 239.5) | 205.0  (179.0, 239.0) | 208.0  (176.0, 244.0) | 0.951 |
|  | Ferritin, ng/dL | 403 | 93% | 190.0  (166.0, 218.0) | 179.0  (157.0, 210.0) | 179.0  (167.0, 197.0) | 179.5  (158.0, 203.5) | 181.0  (157.0, 209.0) | 0.474 |
|  | antiHCV | 6,007 | 0% | 30 (0.93) | 32 (1.31) | 2 (0.83) | 5 (5.88) | 71 (1.18) | 0.001 |
|  | antiHBs | 6,007 | 0% | 983 (30.41) | 98 (4.00) | 11 (4.58) | 9 (10.59) | 1144 (19.04) | <0.001 |

**Table S4** Baseline characteristics of patients included in the actual treatment-pattern analysis by treatment approach (cont.)

| **Outcomes** | **Variables** | **N** | **% Missing** | **Treatment group** | | | | **Total** | **p-value** |
| --- | --- | --- | --- | --- | --- | --- | --- | --- | --- |
|  |  |  |  | **No-PBs** | **CBPBs-CBPBs** | **CBPBs-NCBPBs** | **CBPBs-aluminum** |  |  |
| Bone disorders | **Total number of patients, n (%)** |  |  | 3,658 (51.0) | 3,018 (42.1) | 400 (5.6) | 91 (1.3) | 7,167 (100.0) |  |
|  | **Hospital, n(%)** | 7,167 | 0 |  |  |  |  |  | <0.001 |
|  | Ramathibodi Hospital |  |  | 3,229 (88.27) | 2,046 (67.79) | 256 (64.00) | 32 (35.16) | 5,563 (77.62) |  |
|  | Bhumibol Hospital |  |  | 429 (11.73) | 972 (32.21) | 144 (36.00) | 59 (64.84) | 1,604 (22.38) |  |
|  | **age (years), mean(SD)** | 7,167 | 0 | 48.05 (16.83) | 60.60 (15.97) | 61.42 (14.66) | 50.81 (15.81) | 53.98 (17.46) | <0.001 |
|  | **Female, n(%)** | 7,167 | 0 | 1510 (41.28) | 1,524 (50.50) | 180 (45.00) | 45 (49.45) | 3,259 (45.47) | <0.001 |
|  | **Reimbursement, n(%)** | 7,167 | 0 |  |  |  |  |  | <0.001 |
|  | non-CSMBS |  |  | 2,691 (73.56) | 1,714 (56.79) | 88 (22.00) | 78 (85.71) | 4,571 (63.78) |  |
|  | CSMBS |  |  | 967 (26.44) | 1,304 (43.21) | 312 (78.00) | 13 (14.29) | 2,596 (36.22) |  |
|  | **BMI (kg/m^2^ ), mean(SD)** | 3,041 | 58 | 24.13 (4.73) | 2,4.93 (5.36) | 25.28 (4.86) | 23.21 (4.16) | 24.42 (4.95) | <0.001 |
|  | **Marital status, n(%)** | 7,167 | 0 |  |  |  |  |  | <0.001 |
|  | Single |  |  | 1,180 (32.26) | 703 (23.29) | 73 (18.25) | 33 (36.26) | 1,989 (27.75) |  |
|  | Married |  |  | 2,128 (58.17) | 1,886 (62.49) | 284 (71.00) | 47 (51.65) | 4,345 (60.63) |  |
|  | Divorced/widowed |  |  | 346 (9.46) | 419 (13.88) | 42 (10.50) | 11 (12.09) | 818 (11.41) |  |
|  | Priest |  |  | 0 (0.00) | 2 (0.07) | 0 (0.00) | 0 (0.00) | 2 (0.03) |  |
|  | Unknown |  |  | 4 (0.11) | 8 (0.27) | 1 (0.25) | 0 (0.00) | 13 (0.18) |  |
|  | **Education levels, n(%)** | 3,417 | 52 |  |  |  |  |  | <0.001 |
|  | Primary level or less |  |  | 580 (23.00) | 245 (31.17) | 23 (22.55) | 4 (57.14) | 852 (24.93) |  |
|  | Secondary level |  |  | 760 (30.13) | 170 (21.63) | 9 (8.82) | 1 (14.29) | 940 (27.51) |  |
|  | University level or more |  |  | 1,182 (46.87) | 371 (47.20) | 70 (68.63) | 2 (28.57) | 1,625 (47.56) |  |
|  | **Renal replacement therapy, n(%)** | 7,167 | 0 |  |  |  |  |  | <0.001 |
|  | No RRT |  |  | 2,268 (62.00) | 2,221 (74.46) | 141 (35.52) | 29 (31.87) | 4,659 (65.35) |  |
|  | RRT |  |  | 1,390 (38.00) | 762 (25.54) | 256 (64.48) | 62 (68.13) | 2,470 (34.65) |  |
|  | **Comorbid, n(%)** |  |  |  |  |  |  |  |  |
|  | Hypertension | 7,167 | 0 | 1,803 (49.29) | 2,876 (96.41) | 297 (99.25) | 90 (98.90) | 5,166 (72.46) | <0.001 |

**Table S4** Baseline characteristics of patients included in the actual treatment-pattern analysis by treatment approach (cont.)

| **Outcomes** | **Variables** | **N** | **% Missing** | **Treatment group** | | | | **Total** | **p-value** |
| --- | --- | --- | --- | --- | --- | --- | --- | --- | --- |
|  |  |  |  | **No-PBs** | **CBPBs-CBPBs** | **CBPBs-NCBPBs** | **CBPBs-aluminum** |  |  |
| Bone disorders | Diabetes | 7,167 | 0 | 837 (22.88) | 1,560 (52.30) | 238 (59.50) | 44 (48.35) | 2,679 (37.58) | <0.001 |
|  | Dyslipidemia | 7,167 | 0 | 911 (24.90) | 2,033 (68.15) | 329 (82.25) | 52 (57.14) | 3,325 (46.64) | <0.001 |
|  | CVD | 7,167 | 0 | 477 (13.04) | 901 (30.20) | 185 (46.25) | 28 (30.77) | 1,591 (22.32) | <0.001 |
|  | Gout | 7,167 | 0 | 128 (3.50) | 207 (6.94) | 45 (11.25) | 7 (7.69) | 387 (5.43) | <0.001 |
|  | Liver disease | 7,167 | 0 | 143 (3.91) | 173 (5.73) | 41 (10.33) | 2 (2.20) | 341 (4.76) | 0.002 |
|  | SLE | 7,167 | 0 | 49 (1.34) | 64 (2.12) | 9 (2.27) | 1 (1.10) | 119 (1.66) | 0.139 |
|  | Cancer | 7,167 | 0 | 25 (0.68) | 28 (0.93) | 3 (0.76) | 0 (0.00) | 53 (0.74) | 0.261 |
|  | AIDS | 7,167 | 0 | 6 (0.16) | 23 (0.76) | 2 (0.50) | 0 (0.00) | 30 (0.42) | 0.004 |
|  | GI disorder | 7,167 | 0 | 115 (3.14) | 313 (10.37) | 15 (7.35) | 9 (9.89) | 480 (6.70) | <0.001 |
|  | Chronic pulmonary disease | 7,167 | 0 | 47 (1.28) | 96 (3.18) | 14 (3.50) | 0 (0.00) | 157 (2.19) | <0.001 |
|  | Tumor lysis syndrome | 7,167 | 0 | 2 (0.05) | 0 (0.00) | 0 (0.00) | 0 (0.00) | 2 (0.03) | 0.751 |
|  | Hypoparathyroidism | 7,167 | 0 | 17 (0.46) | 44 (1.46) | 3 (0.75) | 3 (3.30) | 67 (0.93) | 0.282 |
|  | Secondary Hyperparathyroidism | 7,167 | 0 | 275 (7.52) | 833 (27.92) | 195 (48.75) | 54 (59.34) | 1,357 (19.03) | <0.001 |
|  | **Laboratory, median(IQR)** |  |  |  |  |  |  |  |  |
|  | eGFR, ml/min/1.73 m^2^ | 7,155 | 0 | 6.2 (4.2, 17.8) | 6.6 (4.6, 11.4) | 4.2 (3.4, 5.6) | 4.8 (3.6, 6.6) | 6.4 (4.4, 12.6) | <0.001 |
|  | Phosphorus, mg/dL | 7,167 | 0 | 5.6 (5.0, 6.6) | 5.3 (4.8, 6.4) | 6.2 (5.4, 7.4) | 6.6 (6.0, 7.7) | 5.6 (5.0, 6.6) | <0.001 |
|  | PTH, pg/dL | 4,071 | 43 | 322.6  (141.8, 640.6) | 178.0  (89.0, 357.4) | 289.0  (146.0, 634.6) | 313.5  (164.9, 657.6) | 253.0  (115.8, 527.6) | <0.001 |
|  | Calcium, mg/dL | 7,167 | 0 | 9.4  (8.8, 10.0) | 9.3  (8.7, 9.8) | 9.7  (9.1, 10.5) | 9.8  (9.3, 10.5) | 9.4  (8.8, 10.0) | <0.001 |
|  | Hemoglobin, g/dL | 7,167 | 0 | 10.6 (9.1, 11.9) | 9.7 (8.4, 11.1) | 10.9 (9.6, 11.8) | 9.1 (7.4, 10.5) | 10.2 (8.8, 11.5) | <0.001 |
|  | Hematocrit, % | 2,014 | 72 | 29.0  (25.0, 33.0) | 29.0  (24.7, 33.0) | 32.9  (29.8, 36.4) | 28.2  (23.2, 31.6) | 29.1  (25.0, 33.1) | 0.009 |
|  | Albumin, g/dL | 7,167 | 0 | 3.6 (3.2, 4.0) | 3.4 (2.9, 3.8) | 3.7 (3.3, 4.3) | 3.7 (3.2, 4.3) | 3.5 (3.0, 3.9) | <0.001 |
|  | Sodium, mEq/L | 7,127 | 1 | 138.0  (136.0, 140.0) | 138.0  (135.0, 140.0) | 138.0  (136.0, 140.0) | 138.0  (135.0, 140.0) | 138.0  (135.0, 140.0) | <0.001 |

**Table S4** Baseline characteristics of patients included in the actual treatment-pattern analysis by treatment approach (cont.)

| **Outcomes** | **Variables** | **N** | **% Missing** | **Treatment group** | | | | **Total** | **p-value** |
| --- | --- | --- | --- | --- | --- | --- | --- | --- | --- |
|  |  |  |  | **No-PBs** | **CBPBs-CBPBs** | **CBPBs-NCBPBs** | **CBPBs-aluminum** |  |  |
| Bone disorders | Potassium, mEq/L | 3,617 | 50 | 4.6 (4.0, 5.0) | 4.5 (4.1, 5.0) | 4.7 (4.2, 5.1) | 4.7 (4.2, 5.2) | 4.6 (4.1, 5.0) | 0.012 |
|  | Chloride, mEq/L | 7,133 | 0 | 100.0  (97.0, 103.0) | 103.0  (99.0, 107.0) | 99.0  (97.0, 102.0) | 101.0  (97.0, 105.0) | 101.0  (98.0, 105.0) | <0.001 |
|  | Uric, mg/dL | 6,365 | 11 | 7.0 (5.6, 8.5) | 7.8 (6.3, 9.4) | 6.7 (5.6, 8.0) | 7.6 (6.2, 9.1) | 7.4 (5.9, 9.0) | <0.001 |
|  | Glucose, mg/dL | 3,609 | 50 | 101.0  (87.0, 135.0) | 103.0  (88.2, 137.0) | 98.0  (86.0, 133.2) | 104.4  (89.0, 128.0) | 102.6  (88.0, 136.0) | 0.415 |
|  | HbA1C, mg% | 2,501 | 65 | 6.3 (5.6, 7.3) | 6.3 (5.6, 7.3) | 5.8 (5.3, 6.6) | 6.0 (5.5, 7.1) | 6.3 (5.6, 7.3) | 0.018 |
|  | LDL, mg/dL | 4,247 | 41 | 101.8  (79.0, 128.0) | 104.3  (82.0, 133.0) | 94.0  (71.0, 119.0) | 102.0  (78.0, 144.0) | 103.0  (81.0, 131.0) | 0.013 |
|  | Cholesterol, mg/dL | 6,656 | 7 | 179.0  (151.0, 215.0) | 182.0  (150.0, 218.0) | 168.0  (141.0, 199.0) | 184.5  (148.0, 223.0) | 180.0  (150.0, 216.0) | 0.046 |
|  | Triglyceride, mg/dL | 6,157 | 14 | 116.0  (82.0, 158.0) | 128.0  (92.0, 185.0) | 113.0  (80.0, 160.0) | 127.0  (91.0, 178.0) | 122.0  (86.8, 172.0) | <0.001 |
|  | HDL, mg/dL | 4,056 | 43 | 45.0 (37.0, 56.0) | 45.0 (37, 56.0) | 45.0 (36.0, 55.0) | 49.0 (41.0, 56.0) | 45.0 (37.0, 56.0) | 0.897 |
|  | Iron, ug/dL | 2,447 | 66 | 51.0  (35.0, 71.5) | 52.0  (35.9, 73.0) | 58.0  (43.0, 75.0) | 55.0  (42.0, 73.0) | 52.0  (36.0, 73.0) | 0.087 |
|  | TIBC, ug/dL | 2,459 | 66 | 208.9  (173.0, 245.0) | 206.7  (174.0, 245.0) | 209.0  (182.7, 240.0) | 208.6  (184.0, 253.0) | 207.8  (173.7, 245.0) | 0.825 |
|  | Ferritin, ng/dL | 405 | 94 | 188.0  (165.0, 217.0) | 181.0  (156.0, 212.0) | 178.0  (157.0, 202.0) | 192.0  (151.0, 226.0) | 181.0  (157.0, 211.0) | 0.863 |
|  | antiHCV | 7,167 | 0 | 37 (1.01) | 38 (1.26) | 3 (0.75) | 3 (3.30) | 81 (1.13) | 0.244 |
|  | antiHBs | 7,167 | 0 | 1038 (28.38) | 147 (4.87) | 19 (4.75) | 2 (2.20) | 1206 (16.83) | <0.001 |

Abbreviations: Body mass index; (BMI), CBPBs; Calcium-based phosphate binders, CVD; cardiovascular disease, CSMBS; Civil Servant Medical Benefit Scheme, eGFR; estimated Glomerular Filtration Rate, ESRD; End-stage renal disease, HBA1C; Hemoglobin A1C, HDL; High-Density Lipoprotein, HIV; human immunodeficiency virus, IQR; interquartile range, ITT; Intention to treat, GI; Gastrointestinal tract, LDL; Low-Density Lipoprotein, NCBPBs; N; number, Non-calcium-based phosphate binders i.e., lanthanum or sevelamer; OS; overall survival outcome, PBs; Phosphate binders, PPA; Per-protocol analysis, SLE; PTH; Parathyroid hormone levels, RRT, Renal Replacement Therapy, Systemic lupus erythematosus, TIBC; Total iron binding capacity

# Table S5*.* Median (IQR) follow-up time by treatment approach and outcomes

| Treatment approaches | Median (Q1, Q3) follow-up time | | | |
| --- | --- | --- | --- | --- |
|  | No-PBsNo-PBs* | CBPBsCBPBs-CBPBs* | NCBPBsCBPBs-NCBPBs* | Aluminum hydroxideCBPBs-aluminum* |
| *Primary outcome: OS* | | | | |
| ITT | 3.88(1.97, 6.31) | 4.14(1.93, 7.19) | 2.77(1.43, 5.48) | 4.72(1.91, 8.90) |
| PPA | 3.88(1.97, 6.31) | 3.41(1.54, 6.05) | 2.29(0.89, 3.93) | 0.79(0.45, 1.85) |
| Actual treatment-pattern* | 3.88(1.97, 6.31) | 3.77(1.74, 6.69) | 5.14(3.04, 8.28) | 6.36(3.52, 9.24) |
| *Primary outcome: Time to CVD* | | | | |
| ITT | 1.98(0.77, 4.13) | 2.22(0.72, 4.83) | 2.07(0.67, 3.99) | 2.06(0.54, 6.21) |
| PPA | 1.98(0.77, 4.13) | 1.92(0.64, 4.51) | 1.35(0.57, 2.61) | 0.77(0.33, 0.78) |
| Actual treatment-pattern* | 1.98(0.77, 4.13) | 1.79(0.60, 4.34) | 4.16(2.45, 6.49) | 3.84(2.58, 7.10) |
| *Secondary outcome: Time to bone disorders* | | | | |
| ITT | 3.84(1.94, 6.28) | 3.82(1.77, 6.79) | 2.57(1.37, 5.44) | 3.75(1.73, 8.19) |
| PPA | 3.84(1.94, 6.28) | 3.14(1.37, 5.75) | 2.29(0.81, 4.01) | 0.79(0.45, 1.85) |
| Actual treatment-pattern* | 3.84(1.94, 6.28) | 3.56(1.59, 6.35) | 4.88(2.78, 7.90) | 6.17(3.51, 8.74) |

Abbreviations: CBPBs; Calcium-based phosphate binders, CVD; cardiovascular disease, CSMBS; IQR; interquartile range, ITT; Intention to treat, NCBPBs; N; number, Non-calcium-based phosphate binders i.e., lanthanum or sevelamer; OS; overall survival outcome, PBs; Phosphate binders, PPA; Per-protocol analysis, Q; quartile

# Table S6*.* Estimation of PB effects on bone disorder: Parametric survival analysis with Weibull survival distribution (ITT approach)

| **Factors** | **Adjusted HR**  **(95%CI)** | **p-value** |
| --- | --- | --- |
| Treatments |  |  |
| No-PBs | 1 |  |
| CBPBs | 2.639 (2.114, 3.296) | <0.001 |
| NCBPBs | 2.028 (1.102, 3.732) | 0.023 |
| Aluminum hydroxide | 4.113 (2.348, 7.204) | <0.001 |
| Age (years) | 1.014 (1.007, 1.020) | <0.001 |
| Sex |  |  |
| Female | 1 |  |
| Male | 0.644 (0.542, 0.764) | <0.001 |
| RRT |  |  |
| No RRT | 1 |  |
| PD | 0.831 (0.525, 1.315) | 0.429 |
| HD | 1.198 (0.975, 1.468) | 0.084 |
| KT | 1.533 (1.160, 2.034) | 0.003 |
| CVD | 1.390 (1.164, 1.661) | <0.001 |
| Secondary Hyperparathyroidism | 1.555 (1.280, 1.891) | <0.001 |
| Phosphorus, mg/dL | 0.889 (0.840, 0.942) | <0.001 |
| Calcium, mg/dL | 1.195 (1.107, 1.290) | <0.001 |
| Anti-PTH agents | n/a | n/a |
| Reimbursement |  |  |
| Non CSMBS | n/a | n/a |
| CSMBS | n/a | n/a |
| Hospital |  |  |
| Bhumibol Hospitals | n/a | n/a |
| Ramathibodi Hospitals | n/a | n/a |
| Hypertension | n/a | n/a |
| Diabetes | n/a | n/a |
| Dyslipidemia | n/a | n/a |
| Anemia | n/a | n/a |
| Gout | n/a | n/a |

Abbreviations: CBPBs, calcium-based phosphate binders; CI, confidence interval; CSMBS, civil servant medical benefit scheme; CVD, cardiovascular disease; ESRD, end-stage renal disease; HD; Hemodialysis, HR; Hazard ratio, KT; Kidney transplantation; PBs, phosphate binders; PD; peritoneal dialysis, PTH; Parathyroid hormone, RRT, renal replacement therapy.

# Table S7*.* Summary E-value point estimates and confidence intervals by treatment approach: actual treatment-pattern approach

| **Treatments** | | **E-value** for point estimate  **(E***_est_***)** | **E-value** for confidence limit  **(E***_CI_***)** |
| --- | --- | --- | --- |
| **OS** | | | |
| CBPBs-CBPBs | No-PBs | 1.550 | 1.353 |
| CBPBs-NCBPBs |  | 5.741 | 4.715 |
| CBPBs-aluminum |  | 1.710 | 1.524 |
| CBPBs-NCBPBs | CBPBs-CBPBs | 4.448 | 3.642 |
| CBPBs-aluminum |  | 1.291 | 1.000 |
| CBPBs-aluminum | CBPBs- NCBPBs | 4.253 | 3.804 |
| **Time to CVD** | | | |
| CBPBs-CBPBs | No-PBs | 1.865 | 1.218 |
| CBPBs-NCBPBs |  | 2.376 | 1.621 |
| CBPBs-aluminum |  | 1.421 | 1.000 |
| CBPBs-NCBPBs | CBPBs-CBPBs | 3.331 | 2.679 |
| CBPBs-aluminum |  | 1.345 | 1.175 |
| CBPBs-aluminum | CBPBs- NCBPBs | 1.515 | 1.173 |
| **Time to bone disorders** | | | |
| CBPBs-CBPBs | No-PBs | 5.441 | 3.458 |
| CBPBs-NCBPBs |  | 4.195 | 3.138 |
| CBPBs-aluminum |  | 1.991 | 1.378 |
| CBPBs-NCBPBs | CBPBs-CBPBs | 3.108 | 2.482 |
| CBPBs-aluminum |  | 1.541 | 1.326 |
| CBPBs-aluminum | CBPBs- NCBPBs | 1.744 | 1.386 |

Abbreviations: CBPBs; Calcium-based phosphate binders, CI; confidence interval, CVD; cardiovascular disease, est; estimation, IQR; interquartile range, NCBPBs; N; number, Non-calcium-based phosphate binders i.e., lanthanum or sevelamer; OS; overall survival outcome, PBs; Phosphate binders

# Figure S1. Treatment approaches considered

**
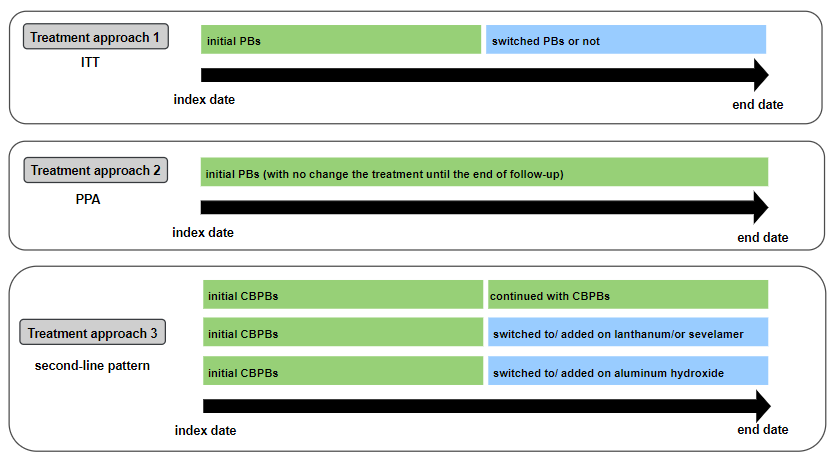
**

# Figure S2. KM curves for all-cause mortality by treatment approach


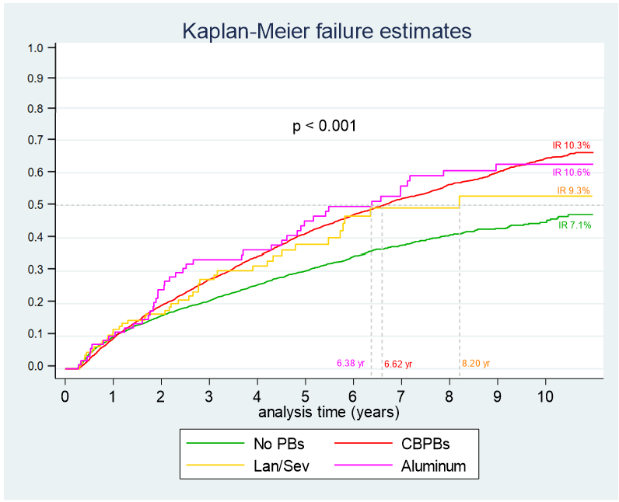
 A) ITT approach


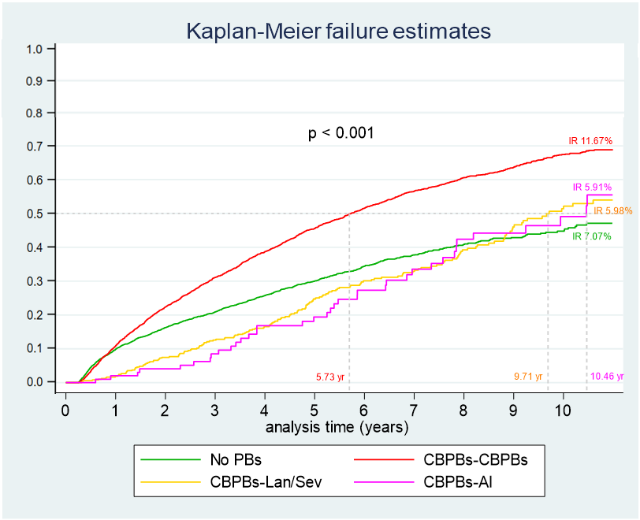

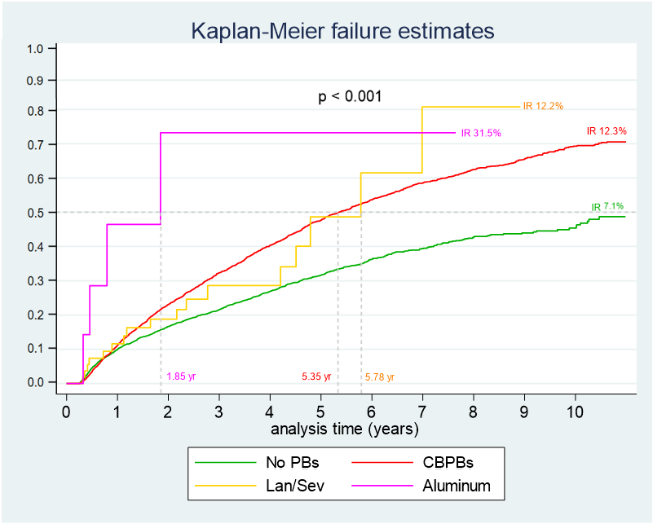
 B) PPA approach C) Actual treatment-pattern approach

# Figure S3. KM curves for CVD events by treatment approach


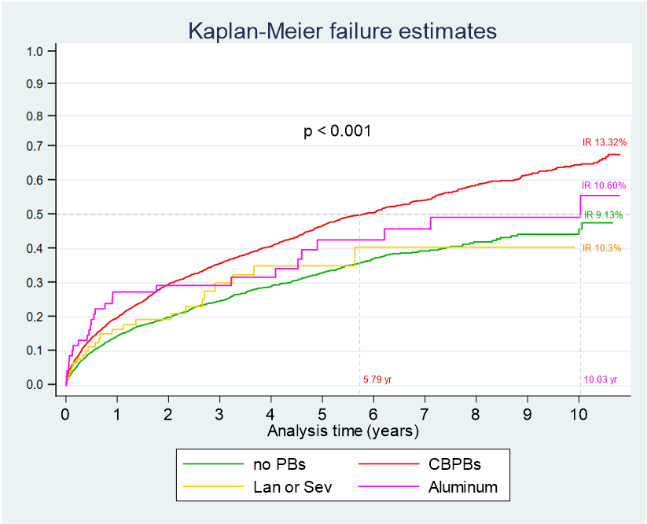
A) ITT approach


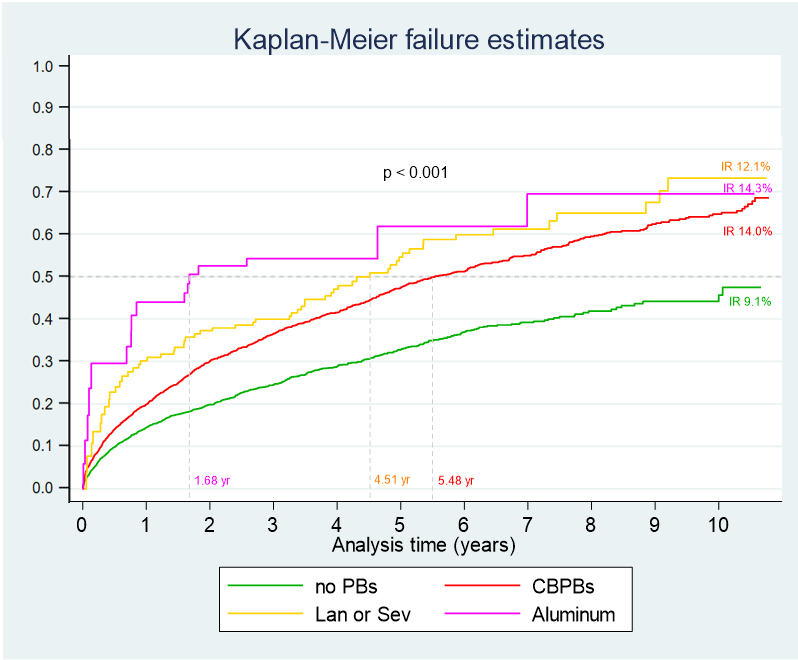

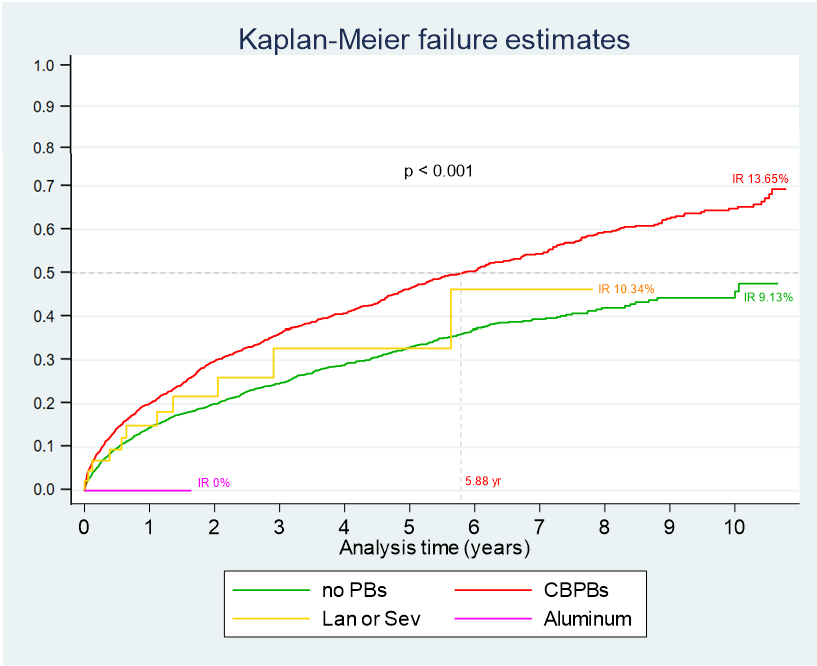
 B) PPA approach C) Actual treatment-pattern approach

# Method S1. Clinical trial emulation and treatment approaches

Real-world End stage renal disease (ESRD) patients with hyperphosphatemia may be eligible for different treatment options predicated on serum phosphate levels, therapeutic goals, availability, and affordability. Treatment option data were retrieved from electronic records. Therefore, three approaches were considered to address variation in the potential prescribing patterns observed, see Figure S1.

1. ***Approach 1: Intention to treat (ITT)***

Data was analyzed on an ITT approach, i.e., patients were initially assigned to treatment groups regardless of adherence levels and/or, change or termination of treatment. Four treatment groups were considered (no-phosphate-binders (PBs) (control group), calcium-based phosphate-binders (CBPBs), lanthanum/or sevelamer, and aluminum hydroxide initiation).

1. ***Approach 2: Per-protocol analysis (PPA)***

This PPA approach considered only patients in receipt of PBs only for the duration of the study period. Therefore, individual patients who switched treatments or were included in another treatment group were excluded from this analysis. Four treatment groups were generated (no-PBs (control group), persistent CBPBs, persistent lanthanum/or sevelamer, and persistent aluminum hydroxide).

1. ***Approach 3: Actual treatment-pattern analysis***

CBPBs are commonly prescribed as the first-line treatment option for patients with ESRD and hyperphosphatemia in line with Thai health policy given the higher costs associated with non-CBPBs or NCBPBs (lanthanum and sevelamer) compared to CBPBs (except aluminum hydroxide). NCBPBs are not reimbursed to healthcare providers unless NCBPBs are recommended as a secondary treatment option if patients have not responded to treatment with CBPBs, or contraindications arise, or alternatively patients are in apposition to pay. As such, this approach differentiates the effectiveness of PBs as an actual treatment pattern when treatment with CBPBs is unsuccessful, which is not an uncommon occurrence.

We focused on patients initially treated with CBPBs and then switched to/or additionally received NCBPBs (i.e., lanthanum/or sevelamer, and aluminum hydroxide) if a target serum phosphate level was not maintained, for comparisons with patients not in receipt of any PBs. Four groups were compared in this approach including no-PBs (control group), CBPBs-CBPBs, CBPBs-lanthanum/or sevelamer, and CBPBs-aluminum hydroxide.

- *No-PB patients (control group)*
- *CBPBs-CBPBs patients* were defined as those were initially treated with CBPBs and did not switch to/or additionally received any other PBs (i.e., lanthanum/sevelamer or aluminum hydroxide), or those received persistent CBPBs treatment in order to maintain serum phosphate levels within the normal range*.*
- *CBPBs-NCBPBs, and CBPBs-aluminum* *hydroxide* were defined as those were initially treated with CBPBs but were then switched to/or additionally received any NCBPBs (i.e., lanthanum or sevelamer), or aluminum hydroxide for a period greater than three months in order to maintain serum phosphate levels within the normal range.

# Method S2 Statistical analysis

1. ***Propensity score (PS) using a counterfactual approach by likelihood-adjusted-censoring with inverse-probability-weighted regression adjustment (LAC-IPWRA)***

Treatment and outcome models were developed using a LAC-IPWRA approach.

**Treatment Model:** A multi-logit model was used to estimate the probability of receiving phosphate binders (PBs). Initial model selection included eleven baseline covariates (age, sex, RRT, reimbursement, serum phosphate, serum calcium, HTN, DM, DLP, CVD, hyperparathyroidism). Covariates with p<0.1 or clinical significance (OR>1.5 or OR<0.67, determined in consultation with nephrologists) were included in a multivariate multi-logit model. Backward elimination with likelihood ratio testing (p<0.05) and clinical significance yielded a final model with ten covariates: age, sex, reimbursement, RRT, serum phosphate, serum calcium, DM, HTN, DLP, hyperparathyroidism. Model assumptions were assessed and adjusted as needed.

| **Variables** | **Univariate analysis** | | **Multivariate analysis** | | | |  |
| --- | --- | --- | --- | --- | --- | --- | --- |
|  | **OR (95%Cl)** | **P-value** | **OR (95%Cl)** | | | **P-value** |  |
| **CBPBs vs No-PBs** | | | | | | |  |
| Age, years | 1.048 (1.045, 1.051) | <0.001 | 1.039 (1.035, 1.043) | | | <0.001 |  |
| Sex |  |  |  | | |  |  |
| Female | 1 |  | 1 | | |  |  |
| Male | 0.635 (0.578, 0.698) | <0.001 | 0.654 (0.581, 0.835) | | | <0.001 |  |
| Reimbursement |  |  |  | | |  |  |
| Non CSMBS | 1 |  | 1 | | |  |  |
| CSMBS | 2.235 (2.025, 2.468) | <0.001 | 1.349 (1.207, 1.508) | | | <0.001 |  |
| RRT |  |  |  | | |  |  |
| No RRT | 1 |  | 1 | | |  |  |
| RRT | 0.550 (0.496, 0.609) | <0.001 | 1.506 (1.345, 1.686) | | | <0.001 |  |
| Phosphorus, mg/dL | 0.911 (0.881, 0.942) | <0.001 | 1.012 (0.987, 1.038) | | | 0.213 |  |
| Calcium, mg/dL | 0.914 (0.875, 0.955) | <0.001 | 0.901 (0.863, 0.940) | | | <0.001 |  |
| Hypertension | 13.112 (8.687, 19.789) | <0.001 | 2.753 (2.445, 5.759) | | | <0.001 |  |
| Diabetes | 3.114 (2.838, 3.416) | <0.001 | 1.614 (1.447, 1.801) | | | <0.001 |  |
| Dyslipidemia | 4.336 (3.907, 4.811) | <0.001 | 1.905 (1.686, 2.152) | | | <0.001 |  |
| Secondary Hyperparathyroidism | 0.846 (0.772, 0.928) | <0.001 | 1.540 (1.378, 1.721) | | | <0.001 |  |
| CVD | 2.818 (2.512, 3.162) | <0.001 | 1.348 (0.996, 1.504) | | | 0.071 |  |
| **NCBPBs vs No-PBs** | | | | | | |  |
| Age, years | 1.046 (1.038, 1.055) | <0.001 | 1.031 (1.017, 1.047) | | | <0.001 |  |
| Sex |  |  |  | | |  |  |
| Female | 1 |  | 1 | | |  |  |
| Male | 0.745 (0.572, 0.969) | 0.028 | 1.596 (0.747, 2.430) | | | 0.639 |  |
| Reimbursement |  |  |  | | |  |  |
| Non CSMBS | 1 |  | 1 | | |  |  |
| CSMBS | 14.934 (10.397, 21.450) | <0.001 | 6.850 (4.201, 11.169) | | | <0.001 |  |
| RRT |  |  |  | | |  |  |
| No RRT | 1 |  | 1 | | |  |  |
| RRT | 4.108 (3.076, 5.487) | <0.001 | 1.567 (1.010, 2.430) | | | <0.001 |  |
| Phosphorus, mg/dL | 1.209 (1.129, 1.295) | <0.001 | | 0.901 (0.813, 0.999) | 0.049 | |  |
| Calcium, mg/dL | 1.411 (1.267, 1.571) | <0.001 | | 1.152 (1.008, 1.319) | 0.039 | |  |
| Hypertension | 4.784 (0.659, 34.682) | 0.121 | | 1.764 (0.411, 7.570) | 0.445 | |  |
| Diabetes | 1.848 (1.107, 3.087) | 0.019 | | 1.138 (0.758, 1.709) | 0.530 | |  |
| Dyslipidemia | 3.942 (1.521, 7.604) | 0.038 | | 1.525 (0.936, 2.485) | 0.090 | |  |
| Secondary Hyperparathyroidism | 3.167 (2.074, 6.307) | <0.001 | | 3.112 (2.096, 4.618) | <0.001 | |  |
| CVD | 1.765 (0.948, 3.286) | 0.073 | | 0.657 (0.433, 1.003) | 0.055 | |  |
| **Aluminum hydroxide vs No-PBs** | | | | | | | |
| Age, years | 1.018 (1.006, 1.030) | 0.003 | | 1.016 (0.998, 1.034) | 0.083 | |  |
| Sex |  |  | |  |  | |  |
| Female | 1 |  | | 1 |  | |  |
| Male | 0.720 (0.482, 1.076) | 0.109 | | 0.771 (0.491, 1.213) | 0.261 | |  |
| Reimbursement |  |  | |  |  | |  |
| Non CSMBS | 1 |  | | 1 |  | |  |
| CSMBS | 3.275 (2.143, 5.006) | <0.001 | | 0.875 (0.523, 1.462) | 0.611 | |  |
| RRT |  |  | |  |  | |  |
| No RRT | 1 |  | | 1 |  | |  |
| RRT | 3.275 (2.143, 5.006) | <0.001 | | 1.484 (0.882, 2.495) | 0.136 | |  |
| Phosphorus, mg/dL | 1.313 (1.200, 1.437) | <0.001 | | 1.374 (1.267, 1.549) | <0.001 | |  |
| Calcium, mg/dL | 1.532 (1.322, 1.775) | <0.001 | | 1.498 (1.223, 1.606) | <0.001 | | |
| Hypertension | 7.891 (0.345, 12.321) | 0.265 | | Could not be estimated | | | |
| Diabetes | 2.969 (1.859, 4.741) | <0.001 | | 2.464 (1.473, 4.121) | 0.001 | | |
| Dyslipidemia | 2.486 (1.504, 4.108) | <0.001 | | 1.563 (0.895, 2.728) | 0.116 | | |
| Secondary Hyperparathyroidism | 1.993 (1.275, 3.115) | 0.002 | | 2.934 (1.829, 4.736) | <0.001 | | |
| CVD | 1.354 (0.755, 2.428) | 0.309 | | 1.086 (0.691, 1.831) | 0.633 | | |

Abbreviations: CBPBs, calcium-based phosphate binders; CI, confidence interval; CSMBS, civil servant medical benefit scheme; CVD, cardiovascular disease; ESRD, end-stage renal disease; OR; Odd ratio; PBs, phosphate binders; RRT, renal replacement therapy.

**Outcome Model:** A parametric survival model (Weibull distribution) was used to estimate treatment effects. Twelve covariates (age, sex, RRT modality, reimbursement, serum phosphate, serum calcium, HTN, DM, DLP, CVD, bone disorders related to ESRD, hyperparathyroidism) were initially considered, using a similar selection process as the treatment model (univariate analysis: p<0.1 or clinical significance [HR>1.5 or HR<0.67]; multivariate model with backward elimination: p<0.05 or clinical significance). The final model retained all twelve covariates. Interactions were explored but did not improve model performance. Adjusted hazard ratios (HRs) with 95% confidence intervals (CIs) were then estimated. (Supplementary Method S2)

| **Factors** | **Crude HR**  **(95%CI)** | **p-value** | **Adjusted HR**  **(95%CI)** | **p-value** |
| --- | --- | --- | --- | --- |
| Treatment groups |  |  |  |  |
| no PBs | 1 |  | 1 |  |
| CBPBs | 1.460 (1.357, 1.570) | <0.001 | 0.960 (0.771, 0.987) | <0.001 |
| NCBPBs | 1.308 (0.958, 1.786) | 0.091 | 0.874 (0.690, 1.296) | 0.728 |
| Aluminum hydroxide | 1.494 (1.109, 2.012) | 0.008 | 1.077 (0.798, 1.455) | 0.626 |
| Age (years) | 1.046 (1.044, 1.049) | <0.001 | 1.039 (1.036, 1.042) | <0.001 |
| Gender |  |  |  |  |
| Female | 1 |  | 1 |  |
| Male | 0.927 (0.864, 0.993) | 0.030 | 1.184 (1.102, 1.273) | <0.001 |
| Reimbursement |  |  |  |  |
| Non CSMBS | 1 |  | 1 |  |
| CSMBS | 1.568 (1.464, 1.681) | <0.001 | 0.924 (0.855, 0.998) | 0.045 |
| RRT |  |  |  |  |
| No RRT | 1 |  | 1 |  |
| PD | 1.273 (1.113, 1.457) | <0.001 | 1.622 (1.412, 1.863) | <0.001 |
| HD | 1.086 (1.006, 1.173) | 0.035 | 1.111 (1.024, 1.206) | 0.011 |
| KT | 0.287 (0.247, 0.335) | <0.001 | 0.721 (0.613, 0.849) | <0.001 |
| Hypertension | 3.064 (2.463, 3.812) | <0.001 | 1.288 (1.023, 1.622) | 0.031 |
| Diabetes | 2.868 (2.663, 3.090) | <0.001 | 1.614 (1.484, 1.755) | <0.001 |
| Dyslipidemia | 1.978 (1.823, 2.145) | <0.001 | 0.881 (0.799, 0.973) | 0.012 |
| CVD | 2.731 (2.543, 2.934) | <0.001 | 1.863 (1.716, 2.022) | <0.001 |
| Bone disorders related ESRD | 1.721 (1.563, 1.895) | <0.001 | 1.241 (1.121, 1.373) | <0.001 |
| Secondary Hyperparathyroidism | 0.450 (0.415, 0.487) | <0.001 | 0.559 (0.514, 0.608) | <0.001 |
| Phosphorus, mg/dL | 1.211 (1.191, 1.231) | <0.001 | 1.273 (1.254, 1.293) | <0.001 |
| Corrected calcium, mg/dL | 1.429 (1.390, 1.469) | <0.001 | 1.406 (1.370, 1.444) | <0.001 |

Abbreviations: CBPBs, calcium-based phosphate binders; CI, confidence interval; CSMBS, civil servant medical benefit scheme; CVD, cardiovascular disease; ESRD, end-stage renal disease; HD, hemodialysis; KT, kidney transplantation; PBs, phosphate binders; PD, peritoneal dialysis; RRT, renal replacement therapy

Potential outcome means (POMs) (i.e., the average time to death/CVD events/bone disorders for each treatment group) were estimated. Additionally, adjusted hazard ratios (HRs) with 95% confidence intervals (CIs) were estimated by weighted parametric survival models.

1. ***Assumptions checking for a treatment model***

Two treatment model assumptions were considered to determine the most appropriate treatment effect model for observational survival-time data:

1. *The conditional independence*

This assumption requires that the potential outcomes should be independent from the treatment assignments conditioning on the covariates. This is analogous to a process of randomization in a randomized controlled trial (RCT). If the treatment model is well specified, it should be able to balance all covariates among treatment groups. This assumption was verified to ensure that treatment assignments after weighting by PS were performed in a manner as efficient as randomization conditioning on the covariates. This assumption can be assessed by estimating the weighted standardized mean difference (SMD) and its variance ratio between the treatment and control groups for each covariate. We expect that the weighted SMD was closed to 0 or ≤ 0.2 and a variance ratio was 1. The density plot of each covariate was also constructed to check balancing after weighting*.* Additionally, the overall balance test was performed using Chi-square.

1. *Treatment overlap assumption* was checked to ensure that each patient has a positive probability of being assigned to each treatment option. This can be checked by plotting a graph of PS score or probability of treatment assignment on the x-axis and a density of propensity score on the y-axis between the treatment and control groups.
2. ***Results of treatment models and assumptions checking***

Ten covariates initially met the model selection criteria: age, sex, reimbursement, RRT, serum calcium, serum phosphate, DM, HTN, DLP, and hyperparathyroidism. However, four covariates were excluded: HTN (due to model convergence issues in the Aluminum hydroxide vs. No-PBs comparison); and DM, DLP, and hyperparathyroidism (due to violations of conditional independence assumptions—standardized mean differences >0.2—and treatment probability overlap, despite assessing interactions). These four were subsequently controlled for in the outcome model. The PS model for each outcome included six significant covariates: age, sex, reimbursement scheme, RRT, serum phosphate level, and serum calcium level (see Table below).

| **Variables** | **Univariate analysis** | | **Multivariate analysis** | | |  |
| --- | --- | --- | --- | --- | --- | --- |
|  | **OR (95%Cl)** | **P-value** | **OR (95%Cl)** | | **P-value** |  |
| **CBPBs vs No-PBs** | | | | | |  |
| Age, years | 1.048 (1.045, 1.051) | <0.001 | 1.044 (1.041, 1.048) | | <0.001 |  |
| Sex |  |  |  | |  |  |
| Female | 1 |  | 1 | |  |  |
| Male | 0.635 (0.578, 0.698) | <0.001 | 0.756 (0.683, 0.837) | | <0.001 |  |
| Reimbursement |  |  |  | |  |  |
| Non CSMBS | 1 |  | 1 | |  |  |
| CSMBS | 2.235 (2.025, 2.468) | <0.001 | 1.253 (1.120, 1.403) | | <0.001 |  |
| RRT |  |  |  | |  |  |
| No RRT | 1 |  | 1 | |  |  |
| RRT | 0.550 (0.496, 0.609) | <0.001 | 0.782 (0.700, 0.874) | | <0.001 |  |
| Phosphorus, mg/dL | 0.911 (0.881, 0.942) | <0.001 | 1.023 (0.987, 1.061) | | 0.213 |  |
| Calcium, mg/dL | 0.914 (0.875, 0.955) | <0.001 | 0.846 (0.807, 0.888) | | <0.001 |  |
| **NCBPBs vs No-PBs** | | | | | |  |
| Age, years | 1.046 (1.038, 1.055) | <0.001 | 1.035 (1.022, 1.049) | | <0.001 |  |
| Sex |  |  |  | |  |  |
| Female | 1 |  | 1 | |  |  |
| Male | 0.745 (0.572, 0.969) | 0.028 | 1.014 (0.691, 1.488) | | 0.943 |  |
| Reimbursement |  |  |  | |  |  |
| Non CSMBS | 1 |  | 1 | |  |  |
| CSMBS | 14.934 (10.397, 21.450) | <0.001 | 5.880 (3.724, 9.286) | | <0.001 |  |
| RRT |  |  |  | |  |  |
| No RRT | 1 |  | 1 | |  |  |
| RRT | 4.108 (3.076, 5.487) | <0.001 | 3.352 (2.271, 4.950) | | <0.001 |  |
| Phosphorus, mg/dL | 1.209 (1.129, 1.295) | <0.001 | 1.238 (1.110, 1.381) | <0.001 | |  |
| Calcium, mg/dL | 1.411 (1.267, 1.571) | <0.001 | 1.484 (1.292, 1.704) | <0.001 | |  |
| **Aluminum hydroxide vs No-PBs** | | | | | | |
| Age, years | 1.018 (1.006, 1.030) | 0.003 | 1.038 (1.024, 1.052) | <0.001 | |  |
| Sex |  |  |  |  | |  |
| Female | 1 |  | 1 |  | |  |
| Male | 0.720 (0.482, 1.076) | 0.109 | 0.737 (0.487, 1.115) | 0.148 | |  |
| Reimbursement |  |  |  |  | |  |
| Non CSMBS | 1 |  | 1 |  | |  |
| CSMBS | 3.275 (2.143, 5.006) | <0.001 | 0.352 (0.195, 0.635) | 0.001 | |  |
| RRT |  |  |  |  | |  |
| No RRT | 1 |  | 1 |  | |  |
| RRT | 3.275 (2.143, 5.006) | <0.001 | 4.010 (2.575, 6.245) | <0.001 | |  |
| Phosphorus, mg/dL | 1.313 (1.200, 1.437) | <0.001 | 1.392 (1.257, 1.541) | <0.001 | |  |
| Calcium, mg/dL | 1.532 (1.322, 1.775) | <0.001 | 1.437 (1.226, 1.685) | <0.001 | | |

Abbreviations: CBPBs, calcium-based phosphate binders; CI, confidence interval; CSMBS, civil servant medical benefit scheme; CVD, cardiovascular disease; ESRD, end-stage renal disease; OR; Odd ratio; PBs, phosphate binders; RRT, renal replacement therapy.

Assumptions checking was shown as follows.

1. Assumption checking for OS

SMDs for each of these covariates ranged from -0.095 to 0.779, -0.061 to 1.101, and 0.050 to 0.919 for CBPBs, NCBPBs, and aluminum hydroxide vs no-PBs, respectively; this indicated a high level of covariate imbalance across treatment groups. After applying PS weighting, the weighted SMDs for the corresponding comparisons were significantly improved, 0.003 to -0.050, 0.010 to 0.429, and 0.028 to 0.378 respectively. Overlapping plots showed positive probabilities for the receipt of each treatment. Results were shown as follows.

- 1. Summary of covariate balance before and after weighting by PS for OS

| Treatment approaches | **Covariates** | **Standardized mean difference (SMD)** | | | | **Variance ratio** | | | | **p-value** | |
| --- | --- | --- | --- | --- | --- | --- | --- | --- | --- | --- | --- |
|  |  | **Before weighted** | | **After weighted** | | **Before weighted** | | **After weighted** | |  |  |
| **ITT** | **CBPBs versus No-PBs** | | | | | | | | | | |
|  | Age | 0.779 | | -0.050 | | 0.875 | | 0.823 | | 0.040 | |
|  | Sex (Male) | -0.213 | | 0.007 | | 1.025 | | 0.999 | | 0.154 | |
|  | CSMBS | 0.480 | | -0.026 | | 1.265 | | 0.990 | | 0.009 | |
|  | Receiving RRT | -0.270 | | 0.029 | | 0.802 | | 1.026 | | 0.015 | |
|  | Serum phosphate | -0.102 | | 0.003 | | 1.232 | | 1.153 | | 0.241 | |
|  | Serum calcium | -0.095 | | 0.035 | | 1.048 | | 1.088 | | 0.011 | |
|  | **NCBPBs versus No-PBs** | | | | | | | | | | |
|  | Age | 0.782 | | 0.029 | | 0.795 | | 0.615 | | 0.071 | |
|  | Sex (Male) | -0.061 | | 0.010 | | 1.024 | | 0.999 | | 0.125 | |
|  | CSMBS | 1.101 | | 0.127 | | 0.946 | | 1.029 | | 0.091 | |
|  | Receiving RRT | 0.463 | | -0.041 | | 1.030 | | 0.961 | | 0.081 | |
|  | Serum phosphate | 0.185 | | 0.121 | | 1.162 | | 0.981 | | 0.046 | |
|  | Serum calcium | 0.654 | | 0.429 | | 0.935 | | 0.669 | | 0.021 | |
|  | **Aluminum hydroxide versus No-PBs** | | | | | | | | | | |
|  | Age | 0.296 | | 0.028 | | 0.688 | | 0.682 | | 0.015 | |
|  | Sex (Male) | -0.138 | | 0.054 | | 1.039 | | 0.994 | | 0.048 | |
|  | CSMBS | 0.144 | | -0.099 | | 1.145 | | 0.956 | | 0.004 | |
|  | Receiving RRT | 0.050 | | 0.069 | | 1.036 | | 1.060 | | 0.002 | |
|  | Serum phosphate | 0.919 | | 0.378 | | 1.565 | | 1.108 | | <0.001 | |
|  | Serum calcium | 0.649 | | 0.259 | | 1.241 | | 0.940 | | <0.001 | |
| **PPA** | **CBPBs versus No-PBs** | | | | | | | | | | |
|  | Age | 0.814 | | -0.033 | | 0.888 | | 0.864 | | 0.036 | |
|  | Sex (Male) | -0.230 | | 0.002 | | 1.023 | | 1.000 | | 0.251 | |
|  | CSMBS | 0.379 | | -0.016 | | 1.252 | | 0.976 | | 0.026 | |
|  | Receiving RRT | -0.284 | | 0.011 | | 0.789 | | 1.010 | | 0.416 | |
|  | Serum phosphate | -0.133 | | 0.006 | | 1.197 | | 1.229 | | 0.080 | |
|  | Serum calcium | -0.099 | | 0.022 | | 1.066 | | 1.099 | | 0.042 | |
| **PPA** | **NCBPBs versus No-PBs** | | | | | | | | | | |
|  | Age | | 0.923 | | 0.153 | | 0.734 | | 0.557 | | 0.046 |
|  | Sex (Male) | | -0.015 | | 0.046 | | 1.023 | | 0.994 | | 0.019 |
|  | CSMBS | | 1.172 | | 0.105 | | 0.891 | | 1.047 | | 0.053 |
|  | Receiving RRT | | 0.327 | | -0.185 | | 1.080 | | 0.817 | | 0.039 |
|  | Serum phosphate | | 0.032 | | 0.220 | | 0.855 | | 1.044 | | 0.011 |
|  | Serum calcium | | 0.717 | | 0.448 | | 0.690 | | 0.554 | | <0.001 |
|  | **Aluminum hydroxide versus No-PBs (excluded from analysis)** | | | | | | | | | | |
| **Actual treatment-pattern approach** | **CBPBs-CBPBs versus No-PBs** | | | | | | | | | | |
|  | Age | | 0.803 | | -0.053 | | 0.890 | | 0.846 | | <0.001 |
|  | Sex | | -0.227 | | 0.004 | | 1.023 | | 1.000 | | 0.251 |
|  | CSMBS | | 0.387 | | -0.028 | | 1.254 | | 0.988 | | 0.093 |
|  | RRT | |  | |  | |  | |  | | 0.0256 |
|  | PD | | -0.178 | | -0.018 | | 0.445 | | 0.919 | |  |
|  | HD | | -0.272 | | -0.030 | | 0.698 | | 1.041 | |  |
|  | KT | | 0.124 | | -0.014 | | 1.873 | | 0.933 | |  |
|  | Serum phosphate | | -0.127 | | 0.004 | | 1.192 | | 1.181 | | 0.474 |
|  | Serum calcium | | -0.096 | | 0.085 | | 1.065 | | 1.105 | | 0.446 |
|  | **CBPBs-NCBPBs versus No-PBs** | | | | | | | | | | |
|  | Age | | 0.774 | | 0.066 | | 0.744 | | 0.789 | | 0.029 |
|  | Sex | | -0.133 | | 0.041 | | 1.028 | | 0.996 | | 0.033 |
|  | CSMBS | | 1.253 | | -0.022 | | 0.809 | | 0.990 | | 0.043 |
|  | RRT | |  | |  | |  | |  | | 0.074 |
|  | PD | | -0.292 | | -0.0004 | | 0.167 | | 0.998 | |  |
|  | HD | | -0.116 | | -0.019 | | 0.882 | | 0.973 | |  |
|  | KT | | -0.047 | | 0.044 | | 0.733 | | 1.218 | |  |
|  | Serum phosphate | | -0.051 | | 0.021 | | 1.356 | | 1.004 | | 0.215 |
|  | Serum calcium | | -0.068 | | -0.008 | | 0.924 | | 0.819 | | 0.158 |
|  | **CBPBs-aluminum versus No-PBs** | | | | | | | | | | |
|  | Age | | 0.169 | | 0.019 | | 0.862 | | 0.839 | | <0.001 |
|  | Sex | | -0.163 | | -0.190 | | 1.038 | | 0.959 | | 0.007 |
|  | CSMBS | | 0.320 | | 0.026 | | 0.626 | | 1.009 | | 0.080 |
|  | RRT | |  | |  | |  | |  | | 0.015 |
|  | PD | | -0.109 | | 0.030 | | 0.648 | | 1.144 | |  |
|  | HD | | -0.294 | | 0.194 | | 0.677 | | 1.184 | |  |
|  | KT | | 0.176 | | -0.060 | | 2.343 | | 0.737 | |  |
|  | Serum phosphate | | 0.264 | | -0.029 | | 2.123 | | 1.042 | | 0.122 |
|  | Serum calcium level | | -0.0145 | | -0.044 | | 1.220 | | 0.815 | | 0.357 |

- 1. Assumptions check after weighting for OS

1. Balance density plot

1. Overlapping plot
2. Assumption checking for CVD

Six covariables were well balanced between treatment comparisons after PS weighting, with weighted SMDs ranging between 0.0002 to 0.079, -0.001 to 0.486, and 0.015 to 0.384 for CBPBs, NCBPBs, and aluminum hydroxide vs no-PBs, respectively. Overlapping plots showed positive probability for equitable allocation of patients across treatment groups.

- 1. Summary of covariate balance before and after weighting by PS for time to CVD events

| Treatment approaches | **Covariates** | **Standardized mean difference (SMD)** | | | | **Variance ratio** | | | | **p-value** | |
| --- | --- | --- | --- | --- | --- | --- | --- | --- | --- | --- | --- |
|  |  | **Before weighted** | | **After weighted** | | **Before weighted** | | **After weighted** | |  |  |
| **ITT** | **CBPBs versus No-PBs** | | | | | | | | | | |
|  | Age | 0.784 | | -0.046 | | 1.002 | | 0.834 | | 0.096 | |
|  | Sex (Male) | -0.225 | | -0.0002 | | 1.023 | | 1.000 | | 0.769 | |
|  | CSMBS | 0.477 | | -0.021 | | 1.384 | | 0.987 | | 0.004 | |
|  | Receiving RRT | -0.321 | | 0.022 | | 0.759 | | 1.019 | | 0.037 | |
|  | Serum phosphate | -0.061 | | -0.008 | | 1.376 | | 1.087 | | 0.124 | |
|  | Serum calcium | -0.229 | | 0.079 | | 1.244 | | 1.207 | | 0.026 | |
|  | **NCBPBs versus No-PBs** | | | | | | | | | | |
|  | Age | 0.887 | | 0.066 | | 0.909 | | 0.642 | | 0.032 | |
|  | Sex (Male) | -0.097 | | -0.041 | | 1.033 | | 1.001 | | 0.039 | |
|  | CSMBS | 1.257 | | 0.167 | | 1.010 | | 1.071 | | 0.019 | |
|  | Receiving RRT | 0.312 | | -0.035 | | 1.069 | | 0.967 | | 0.041 | |
|  | Serum phosphate | 0.150 | | -0.001 | | 1.040 | | 0.622 | | 0.103 | |
|  | Serum calcium | 0.575 | | 0.486 | | 0.854 | | 0.567 | | <0.001 | |
|  | **Aluminum hydroxide versus No-PBs** | | | | | | | | | | |
|  | Age | 0.314 | | -0.050 | | 0.717 | | 0.882 | | 0.046 | |
|  | Sex (Male) | -0.091 | | 0.015 | | 1.036 | | 0.999 | | 0.241 | |
|  | CSMBS | 0.252 | | -0.036 | | 1.285 | | 0.977 | | 0.069 | |
|  | Receiving RRT | 0.112 | | 0.049 | | 1.058 | | 1.040 | | 0.038 | |
|  | Serum phosphate | 1.060 | | 0.384 | | 1.411 | | 1.043 | | <0.001 | |
|  | Serum calcium | 0.706 | | 0.366 | | 1.521 | | 0.947 | | <0.001 | |
| **PPA** | **CBPBs versus No-PBs** | | | | | | | | | | |
|  | Age | 0.807 | | 0.029 | | 1.048 | | 0.899 | | 0.315 | |
|  | Sex (Male) | -0.248 | | -0.006 | | 1.020 | | 1.001 | | 0.412 | |
|  | CSMBS | 0.361 | | -0.008 | | 1.339 | | 0.991 | | 0.269 | |
|  | Receiving RRT | -0.323 | | 0.011 | | 0.757 | | 1.007 | | 0.056 | |
|  | Serum phosphate | 0.949 | | 0.009 | | 0.857 | | 1.156 | | 0.116 | |
|  | Serum calcium | -0.245 | | 0.052 | | 1.257 | | 1.174 | | 0.039 | |
|  | **NCBPBs versus No-PBs** | | | | | | | | | | |
|  | Age | | 1.048 | | 0.194 | | 0.793 | | 0.826 | | 0.044 |
|  | Sex (Male) | | -0.033 | | -0.002 | | 1.031 | | 1.000 | | 0.196 |
|  | CSMBS | | 1.234 | | 0.191 | | 1.039 | | 1.114 | | 0.081 |
|  | Receiving RRT | | 0.069 | | -0.201 | | 1.052 | | 0.986 | | 0.012 |
|  | Serum phosphate | | 0.034 | | 0.175 | | 0.829 | | 0.688 | | <0.001 |
|  | Serum calcium | | 0.612 | | 0.225 | | 0.670 | | 0.926 | | <0.001 |
|  | **Aluminum hydroxide versus No-PBs (excluded from analysis: no event)** | | | | | | | | | | |
| **Actual treatment-pattern approach** | **CBPBs-CBPBs versus No-PBs** | | | | | | | | | | |
|  | Age | | 0.817 | | -0.041 | | 1.012 | | 0.860 | | 0.125 |
|  | Sex (Male) | | -0.237 | | -0.002 | | 1.021 | | 1.0002 | | 0.119 |
|  | CSMBS | | 0.418 | | -0.016 | | 1.366 | | 0.988 | | 0.030 |
|  | Receiving RRT | | -0.317 | | 0.028 | | 0.763 | | 1.022 | | 0.019 |
|  | Serum phosphate | | 0.131 | | -0.015 | | 1.493 | | 1.078 | | 0.072 |
|  | Serum calcium | | -0.236 | | 0.057 | | 1.234 | | 1.024 | | 0.082 |
|  | **CBPBs-NCBPBs versus No-PBs** | | | | | | | | | | |
|  | Age | | 0.836 | | -0.180 | | 0.829 | | 0.766 | | 0.248 |
|  | Sex (Male) | | -0.147 | | 0.122 | | 1.031 | | 0.976 | | 0.115 |
|  | CSMBS | | 1.469 | | -0.079 | | 0.810 | | 0.943 | | 0.053 |
|  | Receiving RRT | | 0.417 | | -0.155 | | 1.036 | | 0.855 | | 0.011 |
|  | Serum phosphate | | 0.227 | | -0.029 | | 1.507 | | 1.019 | | 0.004 |
|  | Serum calcium | | -0.235 | | -0.152 | | 1.205 | | 0.661 | | 0.042 |
|  | **CBPBs-aluminum versus No-PBs** | | | | | | | | | | |
|  | Age | | 0.285 | | -0.092 | | 0.923 | | 0.724 | | 0.025 |
|  | Sex (Male) | | -0.151 | | -0.111 | | 1.039 | | 0.995 | | 0.024 |
|  | CSMBS | | 0.382 | | 0.066 | | 0.481 | | 0.925 | | 0.124 |
|  | Receiving RRT | | 0.304 | | -0.024 | | 1.070 | | 0.980 | | 0.031 |
|  | Serum phosphate | | 0.581 | | 0.121 | | 1.783 | | 0.570 | | 0.002 |
|  | Serum calcium | | -0.277 | | 0.119 | | 1.764 | | 1.147 | | 0.010 |

Abbreviations: CBPBs, calcium-based phosphate binders; CI, confidence interval; CSMBS, civil servant medical benefit scheme; CVD, cardiovascular disease; ESRD, end-stage renal disease; HD; Hemodialysis, ITT; Intention to treat, KT; kidney transplant, OR; Odd ratio; PBs, phosphate binders; PPA; Per-protocol analysis, PD; Peritoneal dialysis, RRT, renal replacement therapy, SMD; standardized mean difference

- 1. Assumptions check after weighting for time to CVD events

1. Balance density plot

1. Overlapping plot
2. Assumption checking for CVD

Six covariables were well balanced between treatment comparisons after PS weighting. Overlapping plots showed positive probability for equitable allocation of patients across treatment groups.

- 1. Summary of covariate balance before and after weighting by PS for time to bone disorder

| Treatment approaches | **Covariates** | **Standardized mean difference (SMD)** | | | | **Variance ratio** | | | | **p-value** | |
| --- | --- | --- | --- | --- | --- | --- | --- | --- | --- | --- | --- |
|  |  | **Before weighted** | | **After weighted** | | **Before weighted** | | **After weighted** | |  |  |
| **ITT** | **CBPBs versus No-PBs** | | | | | | | | | | |
|  | Age | 0.740 | | -0.045 | | 0.886 | | 0.810 | | 0.121 | |
|  | Sex (Male) | -0.173 | | 0.006 | | 1.031 | | 0.999 | | 0.252 | |
|  | CSMBS | 0.445 | | -0.023 | | 1.282 | | 0.989 | | 0.014 | |
|  | Receiving RRT | -0.260 | | 0.026 | | 0.816 | | 1.021 | | 0.231 | |
|  | Serum phosphate | -0.081 | | 0.004 | | 1.276 | | 1.158 | | 0.131 | |
|  | Serum calcium | -0.133 | | 0.042 | | 1.040 | | 1.080 | | 0.030 | |
|  | **NCBPBs versus No-PBs** | | | | | | | | | | |
|  | Age | 0.780 | | 0.045 | | 0.820 | | 0.617 | | 0.089 | |
|  | Sex (Male) | -0.060 | | 0.015 | | 1.027 | | 0.998 | | 0.059 | |
|  | CSMBS | 1.089 | | 0.147 | | 0.989 | | 1.043 | | 0.540 | |
|  | Receiving RRT | 0.473 | | -0.057 | | 1.018 | | 0.949 | | 0.031 | |
|  | Serum phosphate | 0.199 | | 0.128 | | 1.179 | | 0.996 | | 0.025 | |
|  | Serum calcium | 0.642 | | 0.398 | | 0.924 | | 0.662 | | <0.001 | |
|  | **Aluminum hydroxide versus No-PBs** | | | | | | | | | | |
|  | Age | 0.312 | | 0.040 | | 0.716 | | 0.705 | | 0.062 | |
|  | Sex (Male) | -0.123 | | 0.059 | | 1.041 | | 0.988 | | 0.451 | |
|  | CSMBS | 0.177 | | -0.102 | | 1.178 | | 0.945 | | 0.015 | |
|  | Receiving RRT | 0.0617 | | 0.084 | | 1.040 | | 1.066 | | 0.601 | |
|  | Serum phosphate | 0.941 | | 0.391 | | 1.570 | | 1.128 | | 0.001 | |
|  | Serum calcium | 0.643 | | 0.207 | | 1.278 | | 0.947 | | <0.001 | |
| **PPA** | **CBPBs versus No-PBs** | | | | | | | | | | |
|  | Age | 0.775 | | -0.027 | | 0.896 | | 0.853 | | 0.050 | |
|  | Sex (Male) | -0.194 | | 0.001 | | 1.030 | | 1.000 | | 0.564 | |
|  | CSMBS | 0.344 | | -0.128 | | 1.255 | | 0.991 | | 0.002 | |
|  | Receiving RRT | -0.278 | | 0.007 | | 0.799 | | 1.005 | | 0.158 | |
|  | Serum phosphate | -0.121 | | 0.005 | | 1.221 | | 1.233 | | 0.274 | |
|  | Serum calcium | -0.135 | | 0.028 | | 1.048 | | 1.067 | | 0.004 | |
|  | **NCBPBs versus No-PBs** | | | | | | | | | | |
|  | Age | | 0.929 | | 0.150 | | 0.766 | | 0.559 | | 0.165 |
|  | Sex (Male) | | -0.021 | | 0.069 | | 1.026 | | 0.984 | | <0.001 |
|  | CSMBS | | 1.165 | | 0.112 | | 0.930 | | 1.059 | | 0.014 |
|  | Receiving RRT | | 0.360 | | -0.194 | | 1.067 | | 0.871 | | 0.121 |
|  | Serum phosphate | | 0.022 | | 0.268 | | 0.842 | | 1.113 | | 0.001 |
|  | Serum calcium | | 0.687 | | 0.406 | | 0.658 | | 0.535 | | <0.001 |
|  | **Aluminum hydroxide versus No-PBs (excluded from analysis: no event)** | | | | | | | | | | |
| **Actual treatment-pattern approach** | **CBPBs-CBPBs versus No-PBs** | | | | | | | | | | |
|  | Age | | 0.766 | | -0.047 | | 0.897 | | 0.845 | | 0.001 |
|  | Sex (Male) | | -0.192 | | 0.006 | | 1.030 | | 0.999 | | 0.521 |
|  | CSMBS | | 0.360 | | -0.020 | | 1.260 | | 0.989 | | 0.412 |
|  | Receiving RRT | | -0.271 | | 0.023 | | 0.805 | | 1.020 | | 0.124 |
|  | Serum phosphate | | -0.111 | | 0.008 | | 1.217 | | 1.212 | | 0.098 |
|  | Serum calcium | | -0.129 | | 0.025 | | 1.048 | | 1.032 | | 0.113 |
|  | **CBPBs-NCBPBs versus No-PBs** | | | | | | | | | | |
|  | Age | | 0.700 | | 0.074 | | 0.754 | | 0.794 | | 0.429 |
|  | Sex (Male) | | -0.075 | | 0.011 | | 1.023 | | 0.998 | | 0.151 |
|  | CSMBS | | 1.204 | | -0.021 | | 0.884 | | 0.989 | | 0.096 |
|  | Receiving RRT | | -0.214 | | -0.019 | | 0.858 | | 0.983 | | 0.312 |
|  | Serum phosphate | | 0.003 | | 0.0003 | | 1.492 | | 1.004 | | 0.825 |
|  | Serum calcium | | -0.110 | | -0.005 | | 0.962 | | 0.840 | | 0.165 |
|  | **CBPBs-aluminum versus No-PBs** | | | | | | | | | | |
|  | Age | | 0.164 | | -0.049 | | 0.878 | | 0.736 | | 0.279 |
|  | Sex (Male) | | -0.163 | | -0.149 | | 1.042 | | 0.995 | | 0.040 |
|  | CSMBS | | -0.306 | | -0.071 | | 0.635 | | 0.958 | | 0.251 |
|  | Receiving RRT | | -0.224 | | 0.119 | | 0.855 | | 1.091 | | 0.002 |
|  | Serum phosphate | | 0.262 | | -0.048 | | 2.238 | | 1.035 | | 0.653 |
|  | Serum calcium | | -0.174 | | 0.045 | | 1.268 | | 0.809 | | 0.124 |

Abbreviations: CBPBs, calcium-based phosphate binders; CI, confidence interval; CSMBS, civil servant medical benefit scheme; CVD, cardiovascular disease; ESRD, end-stage renal disease; HD; Hemodialysis, ITT; Intention to treat, KT; kidney transplant, OR; Odd ratio; PBs, phosphate binders; PPA; Per-protocol analysis, PD; Peritoneal dialysis, RRT, renal replacement therapy, SMD; standardized mean difference

- 1. Assumptions check after weighting for time to bone disorder

A) Balance density plot

B) Overlapping plot

# Method S3 Sensitivity analysis

1. ***Sensitivity analysis for unmeasured confounders***

E-value was reported to assess the treatment effects on unmeasured confounders in real-world study data. E-value is defined as the minimum strength of association that an unmeasured confounder would need to have with both the treatment assignment and the outcome to fully explain a specific treatment-outcome association, conditional on the measured covariates.

The E-value is computed on the risk ratio (RR) scale, so results of statistical models other than the RR must be converted to the RR scale. The treatment effects on average treatment effects (ATE) and number of patients for each treatment groups after weighting, were used to estimate SMD using *esizeregi*. The SMD was converted to the RR scale to estimate an E-value, as follows:

| *Approximation RR and CIs:* $RR\approx e^{[0.91 \times SMD]}$ $Lower limit (LL) for the RR\approx e^{[0.91\times SMD-1.78\times SD]}$ $Upper limit (UL) for the RR\approx e^{[0.91\times SMD+1.78\times SD]}$ *E-value for the point estimate and CI:*  *If RR > 1:* $E-value \left( point estimate \right)=RR + \sqrt{RR\times(RR-1)}$ $E-value \left( \mathrm{LL} \right)=1 if LL \leq1, else LL + \sqrt{LL\times(LL-1)}$ *If RR < 1:* $E-value \left( point estimate \right)=1/RR + \sqrt{1/RR\times(1/RR-1)}$ $E-value \left( \mathrm{UL} \right)=1 if UL \geq1, else 1/UL + \sqrt{1/UL\times(1/UL-1)}$ |
| --- |

E-values for the point estimate and CI indicate the magnitude of unmeasured confounding required to cause a shift in the treatment estimation. If the E-value for a point estimate and CI are large, this indicates a weak effect of unmeasured confounding. If the E-value for a point estimate and CI are small, it is possible that unmeasured confounding is influencing the treatment effects observed.

1. ***Sensitivity analysis by recalculated PS***

For OS, we recalculated PS by accounting for comorbidities (i.e., DM, HT, DLP, secondary hyperparathyroidism) which were not included in primary analysis but would have been clinically meaningful for treatment allocation. The multivariate multi-logit model included age, sex, reimbursement scheme, RRT modality, serum phosphate, serum calcium levels, DM, HT, DLP, and secondary hyperparathyroidism. A single PS was then recalculated using this final model for death outcomes. The results are as follows.

| **Treatments** | **OS** | |
| --- | --- | --- |
|  | **Mean OS**  **(Primary analysis)** | **Mean OS**  **(sensitivity analysis)** |
| **ITT approach** | | |
| No-PBs | 11.17 (9.57, 12.76) | 11.45 (9.01, 13.89) |
| CBPBs | 11.45 (10.03, 12.87) | 11.48 (9.94, 13.02) |
| NCBPBs | 28.24 (-22.58, 79.06) | 40.12 (-16.34, 96.59) |
| Aluminum hydroxide | 7.26 (3.33, 11.18) | 7.45 (3.20, 11.69) |
| **PPA approach** | | |
| No-PBs | 10.13 (9.14, 11.07) | 10.41 (9.13, 11.70) |
| CBPBs | 10.68 (8.98, 11.59) | 10.69 (7.46, 11.89) |
| NCBPBs | 12.38 (-27.21, 45.22) | 109.52 (-274.19, 493.22) |
| Aluminum hydroxide | excluded | excluded |
| **Actual treatment-patterns approach** | | |
| No-PBs | 10.42 (9.94, 11.20) | 10.30 (9.17, 12.82) |
| CBPBs-CBPBs | 10.86 (8.46, 11.96) | 10.81 (9.73, 11.87) |
| CBPBs-NCBPBs | 13.49 (10.03, 16.93) | 12.80 (8.82, 16.78) |
| CBPBs-Aluminum | 11.01 (7.54, 14.48) | 12.18 (8.45, 15.91) |

**Sensitivity analysis of estimated treatment effects on mean survival by treatment group for all approaches: LAC-IPWRA**

A sensitivity analysis including HT, DM, DLP and hyperparathyroidism yielded largely consistent results, except for increased uncertainty in mean survival time estimates for NCBP treatment in ITT (28.24 [-22.58, 79.06] vs. 40.12 [-16.34, 96.59]) and PPA (12.38 [-27.21, 45.22] vs. 109.52 [-274.19, 493.22]) analyses, likely due to insufficient overlap in treatment probabilities.
